# Computational design of mechanically coupled axle-rotor protein assemblies

**DOI:** 10.1126/science.abm1183

**Published:** 2022-04-21

**Authors:** A. Courbet, J. Hansen, Y. Hsia, N. Bethel, YJ. Park, C. Xu, A. Moyer, S.E. Boyken, G. Ueda, U. Nattermann, D. Nagarajan, D. Silva, W. Sheffler, J. Quispe, A. Nord, N. King, P. Bradley, D. Veesler, J. Kollman, D. Baker

**Affiliations:** 1 Department of Biochemistry, University of Washington, Seattle, USA; 2 Institute for Protein Design, University of Washington, Seattle, USA; 3 Howard Hughes Medical Institute, University of Washington, Seattle, USA; 4 Division of Life Science, The Hong Kong University of Science and Technology, Clear Water Bay, Kowloon, Hong Kong; 5 Monod Bio, Inc, Seattle, USA; 6 Centre de Biologie Structurale (CBS), INSERM, CNRS, Université Montpellier, Montpellier, France; 7 Division of Public Health Sciences, Fred Hutchinson Cancer Research Center, Seattle, USA

## Abstract

Natural molecular machines contain protein components that undergo motion relative to each other. Designing such mechanically constrained nanoscale protein architectures with internal degrees of freedom is an outstanding challenge for computational protein design. Here we explore the *de novo* construction of protein machinery from designed axle and rotor components with internal cyclic or dihedral symmetry. We find that the axle-rotor systems assemble *in vitro* and *in vivo* as designed. Using cryoelectron microscopy we find that these systems populate conformationally variable relative orientations reflecting the symmetry of the coupled components and the computationally designed interface energy landscape. These mechanical systems with internal degrees of freedom are a step towards the design of genetically encodable nanomachines.

## One-Sentence Summary

Computationally designed self-assembling axle-rotor protein systems populate multiple rotational states.

Intricate protein nanomachines in nature have evolved to process energy and information by coupling biochemical free energy to mechanical work. Among the best studied and most sophisticated are protein rotary machines such as the F_1_ motor of ATPase or the bacterial flagellum, which contain axle-like and ring-like symmetric protein components capable of constrained dynamic motion relative to each other (1,2,3). Feynman’s 1959 lecture on nanotechnology as a means to leverage the properties of materials at the molecular scale(4) inspired interest in synthetic nanomachines(5,6). Synthetic chemists were the first to design molecules with mechanically coupled components(7–9). Nucleic acid nanotechnologies have more recently been used to construct rotary systems(10). Designed dynamic protein mechanical systems are of great interest given the richer functionality of proteins, but with this functionality comes more complex folding and a greater diversity of non-covalent interactions which, despite recent advances in design of static protein nanostructures(11–19), has made the design of protein machines an outstanding challenge(20).

**Fig. 1:**
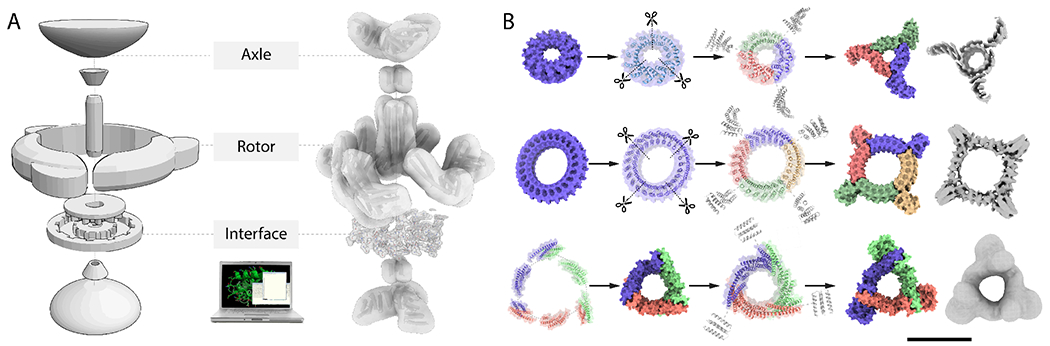
Overview of protein machine assembly and rotor component design approaches. (**A**) (Left) A blueprint of a simple two component machine consisting of an assembly of an axle and a rotor mechanically constrained by the shape of the interface between the two; (Middle) Systematic generation by computational design of a structurally diverse library of machine components and design of interfaces between axle and rotor that mechanically couple the components and direct assembly; (Right) Example of hierarchical design and assembly of a protein machine from axle and rotor components, here a D3 axle and C3 rotor, and interacting interface residues. Wheel-like cyclic DHRs are fused to the end of the axle and rotor components to increase mass, provide a modular handle and a structural signature to monitor conformational variability. (**B**) Hierarchical design strategies for rotor components (Top) A single chain C1 symmetric and internally C12 symmetric alpha-helical tandem repeat protein is split into three subunits, and each is fused to DHRs via helical fusion (HelixFuse) to generate a C3 rotor (C3_R1) with an internal diameter of 28Å. The 6.0Å cryoEM electron density (shown in grey) shows agreement with the design model (monomer subunits colored by chain); (Middle) A single chain C1 symmetric and internally C24 symmetric alpha-helical tandem repeat protein is split into 4 subunits and each is fused to DHRs to generate a C4 rotor (C4_1) with an internal diameter of 57Å. The 7.9Å cryoEM electron density (shown in grey) shows agreement with the design model (monomer subunits colored by chain); (Bottom) Heterooligomeric helical bundles and DHRs are fused and assembled into a higher-ordered closed C3 structure through helical fusion, after which another round of helical fusion protocol is used to fuse DHRs to each subunit, to generate a C3 rotor (C3_R3) with an internal diameter of 41Å. The negative stain electron density (shown in grey) shows agreement with the design model (monomer subunits colored by chain). Scale bar: 10nm

We explored the design of protein mechanical systems through a first-principle, bottom-up approach that decouples operational principles from the complex evolutionary trajectory of natural nanomachines. Previous two component protein assembly design studies have focused on nanomaterials such as icosahedral nanocages(21) and two dimensional arrays(19) in which the components have fixed orientations relative to one another. Here we seek to design a nanoscale *simple machine* or kinematic pair (22,23) in which two protein components can move relative to one another, as a proof of concept of mechanically constrained heterooligomeric assembly that can undergo brownian diffusion along internal degrees of freedom (DOF). We used a hierarchical design approach with three steps: (*i*) *de novo* design of stable and rigid protein components suitable for assembly into constrained mechanical systems (*ii*) directed self-assembly of these components into hetero-oligomeric complexes, (*iii*) shaping of the multistate energetic landscape along the mechanical degrees of freedom. A major challenge is to design the interface between the two designed rigid bodies to have sufficiently low energy to drive self assembly, while still allowing relative motion of components. We started from a machine blueprint that consists of two coupled structural components resembling an axle and rotor (fig. 1A), in which, similar to natural protein rotary systems, the features of the energy landscape are determined by the symmetry of the interacting components, their shape complementarity and specific interactions across the interface.

## Computational design of protein mechanical components

We first sought to design ring-like protein topologies, or rotors, with a range of inner diameter sizes capable of accommodating an axle-like binding partner (fig. 1B). In a first approach we started from *de novo* alpha-helical tandem repeat proteins (24), and redesigned them to form C1 single chain structures or symmetric C3 or C4 homooligomers. In a second approach we started from *de novo* helical repeat proteins (DHRs) and helical bundle heterodimers and used a hierarchical design procedure based on architecture-guided rigid helical fusion(14) to build C3 and C5 cyclic symmetric rotor structures. To facilitate subsequent microscopy characterization and modularity, we fused another set of DHRs at the outer side of the rotors, generating arm-like extensions (fig. 1A–B). Synthetic genes encoding these rotor designs (12xC3s, 12xC4s, 2xC5s) were synthesized and the proteins expressed in *E. coli*. All designed proteins were soluble after purification on nickel-nitrilotriacetic acid (Ni-NTA) columns and ~23% (6/26) had size exclusion chromatography (SEC) profiles that matched the expected theoretical elution profile for the oligomerization state (fig. S1–2, Table S1). These designs were further examined using small-angle X-ray scattering (SAXS)(25,26), negative stain electron microscopy or cryoelectron microscopy (cryoEM) (fig. S1). For the C3_R1 rotor, SAXS data analysis was consistent with the computational model (Volatility ratio (Vr)=4.684, Table S2, fig. S2) and we were able to determine using cryoEM a 6.0Å 3D reconstruction which was close to the design model (backbone RMSD=3.451Å, fig. 1B, fig. S1, fig. S4–5, Table S3). Similar results were obtained for another design of the same topology (C3_R2) (fig. S1). For the C4 design C4_1 we obtained a 7.9Å cryo electron density map closely consistent with the design model (backbone RMSD=1.8Å, fig. 1B, fig. S1, fig. S5–6, Table S3). C3 and C5 rotors with larger inner diameter and different topology (C3_R3, C5_2) were characterized using negative stain EM, yielding low resolution 3D reconstructions consistent with the design model (fig. 1B, fig. S1).

**Fig. 2:**
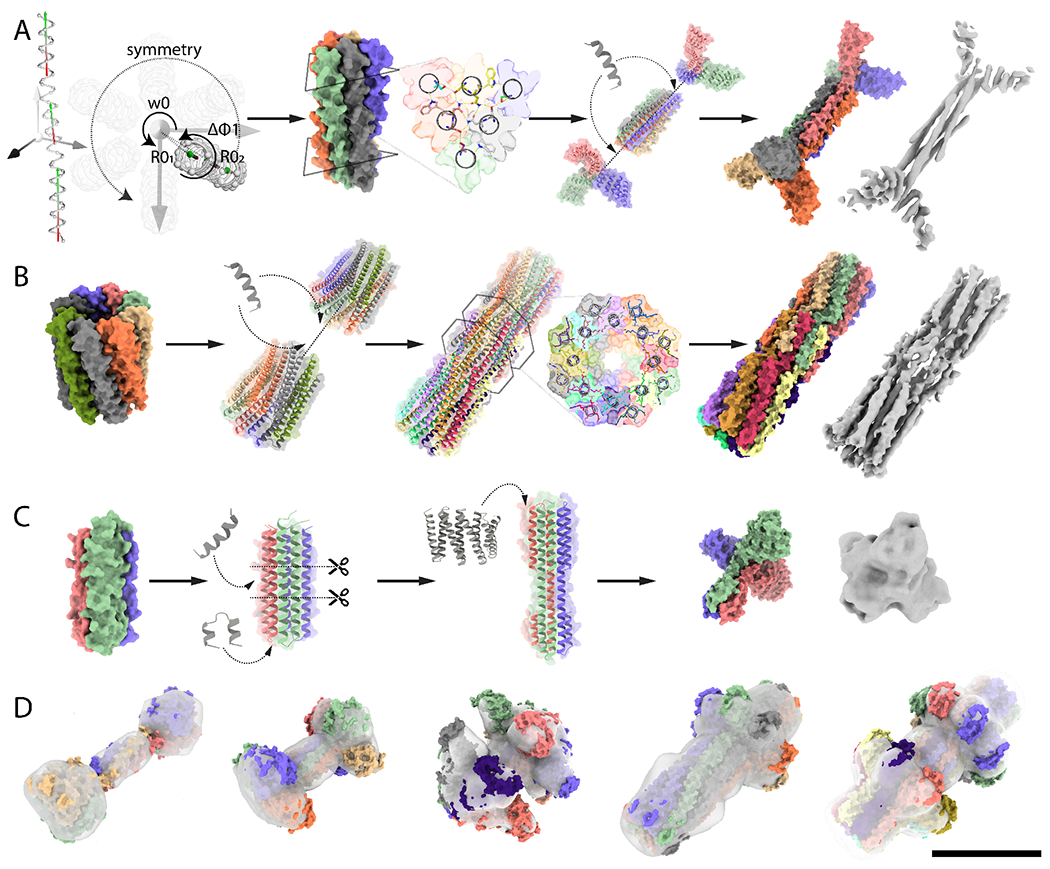
Design of axle machine components. (**A**) Hierarchical design of a D3 symmetric homohexamer axle (D3_3). Parametric design of interdigitated helices in D3 symmetry is achieved by sampling supercoil radius (R_1_,R_2_), helical phase (Δφ_1-1_, Δφ_1-2_), supercoil phase (Δφ_0-1_,Δφ_0-2_) of two helical fragments, and the *z*-offset (Z_off_ and supercoil twist (ω_0_). The interface is designed using the HBNet protocol to identify hydrogen-bond networks spanning the 6 helices mediating high-order specificity. The design is then fused to C3 wheel-like homotrimers using RosettaRemodel. The 4.2Å cryoEM electron density is consistent with the design model (**B**) Hierarchical design of a D8 axle (D8_1). Interdigitated helical extensions at the termini of a parametrically designed C8 homohexamer are sampled using Rosetta BluePrintBuilder and hydrogen bond networks are identified using HBnet, while sampling rotation and translation in D8 symmetry using Rosetta SymDofMover. The 7.4Å cryoEM electron density is in close agreement with the design model; (**C**) Hierarchical design of a C3 homotrimer axle (C3_A1). A parametrically designed C3 homotrimer was circularly permutated and an extra heptad repeat added to increase the aspect ratio, after DHRs were fused to each subunit using Hfuse. The negative stain electron density is consistent with the design model (**D**) Additional axle components overlaid with experimental negative stain electron density, corresponding to D2 (D2_2), D4 (D4_2), D5 (D5_2), C8 (C8_1) and D8 (D8_3) designs. Model monomer subunits are colored by chain, and electron densities are shown as grey surfaces. Scale bar: 10 nm

We next sought to design high aspect ratio protein components, or *axles*, onto which the designed rotor protein could be threaded, using three different design approaches. In a first approach, single helix backbones were parametrically generated and the sequence was optimized in D2, D3 or D4 dihedral symmetry using buried hydrogen bond networks and hydrophobic contacts to produce self-assembling homooligomer interfaces with the high level of specificity needed for dihedral assembly (fig. 2A). To increase the total mass and diversify the shape for subsequent EM analysis, the termini of these rod shape structures were rigidly fused to cyclic homooligomers of matching symmetry (*i.e.* Dn dihedral assemblies were fused with Cn cyclic assemblies) to create dumbbell shaped structures. In a second approach, two copies of designed cyclic homooligomers were assembled into dihedral structures by connecting them with rigid helical bundle connectors built using fragment sampling (fig. 2B). In a third approach, parametrically generated homotrimer backbones consisting of helical hairpin monomer topologies(27) were circularly permuted and elongated to generate extended C3 homooligomers (fig. 2C). Details of the methods, as well as scripts for carrying out the design calculations, are provided in the supplementary materials. Synthetic genes encoding axle designs generated from the three approaches (12xC3s, 12xC5s, 12xC8s, 6xD2s, 12xD3s, 6xD4s, 6xD5s, 12xD8s) were obtained and the proteins were expressed in *E. coli*. The designed proteins that were well-expressed, soluble, and readily purified by Ni-NTA affinity chromatography were further purified on SEC. Success rates for the first, second and third approach were 37.5% (6/16), 43% (14/32) and 33% (4/12) respectively as assessed by the match between estimated molecular weight (MW) from SEC with the MW of the design model (fig. 2D, fig. S1–3, Table S1). Designs with matching SEC traces were further examined using SAXS, negative stain electron microscopy, and cryoEM (fig. S1–3).

The first approach generated D2, D3 and D4 axle-like structures with folds featuring interdigitated helices with extended hydrogen bond networks. We obtained a 4.2Å 3D reconstruction of a D3 axle (D3_3) with backbone nearly identical to the design model (backbone RMSD=1.9Å, fig. 2A, fig. S3,-4, fig. S7); SAXS data were also consistent with the design model (Vr=6.0, Table S2, fig. S1–2). The central homohexameric 50 residue helices (D3_2) could also be solubly expressed and formed an oligomeric self-assembly that eluted at the expected volume (fig. S3, Table S1). D3 design D3_1 consisting of 36 residue long single helices was produced by chemical peptide synthesis and assembled into a homohexamer ( fig. S3, fig. S8), and fusion to wheel-like C3s generated a larger D3 oligomer as designed (D3_4, fig. S3). A D4 peptide homo-oligomer designed using the same approach (D4_1) had a SEC profile consistent with the expected oligomeric state (fig. S2–3, Table S1). Negative stain EM of a D2 design (D2_2) yielded a low resolution 3D reconstruction with the overall features of the design model (fig. 2D, fig. S3); the corresponding central 50 residue D2 peptide (D2_1) could also be expressed and the SEC elution volume corresponded to the expected oligomeric state (fig. S3, Table S1).

The second approach generated D3, D4, D5 and D8 axle-like structures with interdigitated helices with internal cavities in the D5 and D8 cases where each central helix only contacts the two neighboring ones (fig. 2B). We obtained a 7.4Å electron density map of a D8 design (D8_1) revealing a backbone structure nearly identical to the design model (backbone RMSD=2.9Å, fig. 2B, fig. S3, fig. S5–6). This cylinder-shaped homodecahexamer has a large central cavity, an 84 residue helix, and opposing N and C termini close to its center (fig. 2B, fig. S3). Negative stain EM 3D reconstructions of D8_2 and D8_3, D5_2 and D4_2 were consistent with the design models (fig. 2D, fig. S3). We converted several of these designs from dihedral to cyclic symmetry by connecting N and C termini, and two such designs, one C5 (C5_1) and one C8 (C8_1), yielded EM reconstructions with good agreement with the design model (fig. 2D, fig. S1, fig. S3). SAXS profiles of additional designs (4xD3s, 2xD4s and 1xD5) were consistent with the design models with Vr < 10 in most cases and measured MW within 15% of design model for D3_1, D3_8, and within 1% for D5_1 (Table S2, fig. S2–3).

The third approach yielded four C3 axles with smaller aspect ratios and overall sizes, containing a large wheel-like feature at one end, a narrow central three helix section and a six helix section at the other end. SAXS profiles together with SEC traces suggested that the designed oligomerization state is realized in solution (Vr~12, Table S1–2, fig. S1–2). For design C3 A1 we obtained a low resolution cryoEM map that recapitulates the general features of the design model, with prominent C3 symmetric DHR extremities and opposing prism-like extensions (fig. 2C, fig. S1, fig. S4).

## Design of mechanically coupled axle-rotor assemblies

We next investigated the construction of mechanically constrained axle-rotor assemblies from the designed axles and rotors. As noted above, an inherent challenge for the *de novo* design of dynamic protein complexes is to incorporate sufficient energetically favorable interactions to enable directed self-assembly without creating deep energy minima that lock the assembly into a single state and prevent Brownian diffusion along the mechanical DOFs. We explored three approaches for constructing axle-rotor assemblies which result in interfaces with widely varying energetics, shape complementarity, and symmetry.

**Fig. 3:**
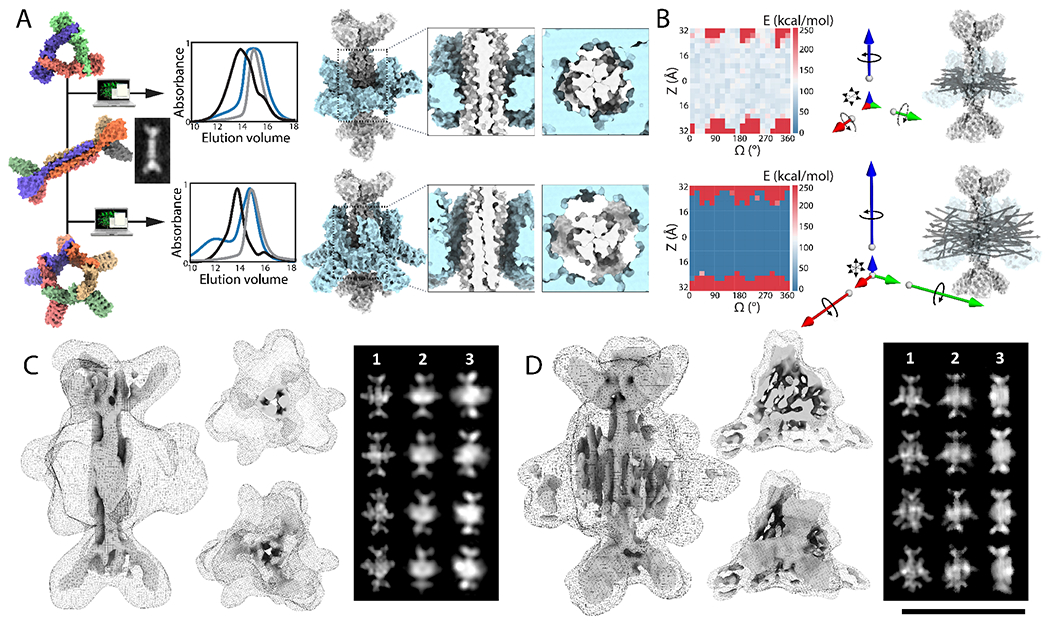
Design of symmetry mismatched D3-C3 and D3-C5 axle-rotor assemblies. (**A**) From Left to right: Models of a D3 axle (D3_3), and C3 (C3_R3) and C5 (C5_2) rotors and cryoEM 2D average of axle alone before assembly. Overlaid SEC chromatograms (absorbance at 215 nm) of axle (grey), rotor (blue), and full assembly (black). Models of D3-C3 and D3-C5 assemblies with top-view and side-view close-up on interfaces: shape and symmetry results in different DOFs. (**B**) (Left) 2D Rotation-Translation energy landscapes showing a large area of low energy where the rotor can be positioned on the axle (REU: Rosetta Energy Units) (Right) MD simulations results are shown as vectors whose magnitude corresponds to the computed mean square displacement of the rotor relative to the axle along the 6 DOFs. The D3-C3 system is largely constrained to rotation along the z axis (blue), while the D3-C5 assembly allows rotation along x (green), y (red) and z, and translation in z, x and y. N-C termini unit vectors of an ensemble of MD trajectories is superimposed on an axle-rotor model structure. (**C**) (Left): 3D CryoEM reconstruction of D3-C3, processed in D3 at 7.8Å resolution suggests that the rotor sits midway across the D3 axle consistent with the designed mechanical DOF. The maps are shown as side view, end-on views and transverse slices, as surface for the axle and as mesh for the rotor, at two different thresholds. (Right): simulated 2D class averages without (1) and with (2) conformational variability, and experimental averages (3). (**D**) (Left) 3D CryoEM reconstruction of D3-C5, processed in C1 at 8.6Å has the overall features of the designed structure, shown as surface and mesh at different thresholds. The 2D averages capture secondary structure corresponding to the C5 rotor, but could not be fully resolved, consistent with the rotor populating conformationally variable states. (Right): simulated 2D class averages without (1) and with (2) conformational variability, and experimental averages (3). Scale bar for cryoEM density: 10nm

First, we sought to construct two-component assemblies in which the rigid body orientation of the axle and rotor was minimally constrained. We designed symmetry mismatched axle-rotor interfaces with low orientational specificity and loose interface packing, allowing only small numbers of close contacts across the interface and employing primarily electrostatic interactions between rotor and axle, which are longer range and less dependent on shape matching than the hydrophobic interactions generally used in protein design. To prevent potential disassembly at low concentrations due to weak axle-rotor interactions, we sought to kinetically trap the rotor around the axle by installing disulfide bonds at the rotor subunit-subunit interfaces. To gain stepwise control on the *in vitro* assembly process, we introduced buried histidine mediated hydrogen bond networks at the asymmetric interfaces between rotor subunits to enable pH controlled rotor assembly (fig. S9, see methods). To test this approach we selected three of the machine components described above -- a D3 axle, a C3 rotor and a C5 rotor -- and constructed axle-rotor assemblies with D3-C3 and D3-C5 symmetries (design D3-C5 and D3-C3 respectively, fig. 3A, fig. S10). To thread axles and rotor together, we computationally sampled rotational and translational DOF, and designed complementary electrostatic interacting surfaces excluding positively charged residues on the axle (Lysine and Arginine) and negatively charged residues (Aspartate and Glutamate) on the rotor. Due to the shape complementarity between the internal diameter of the rotors and the axle thickness, the interface is tight for D3-C3, constraining the rotor on the axle, and loose for D3-C5: by design, the D3-C3 can rotate and translate along the main symmetry axis (z), while the D3-C5 rotor has rotation and translation components along x, y and z (fig. 3A–B, fig. S11). Synthetic genes encoding the one axle and two rotor designs were obtained and the proteins were separately expressed in *E. coli* and purified by Ni-NTA affinity chromatography and SEC, which indicated that the surface redesign did not affect solubility or oligomerization state (fig. S1, fig. S3). Following stoichiometric mixing of the designed D3 axle and C3 rotor, EM analysis showed a collection of assembled and isolated axle and rotor molecules (fig. S9A, **top panel**). After dropping the pH and reducing the disulfide, the particles appeared as a mixture of opened, linear and hard to distinguish particles (fig. S9A, **middle panel**). After restoring the pH under oxidizing conditions, the particles appeared fully assembled by EM (fig. S9A, **bottom panel**). Biolayer interferometry assays showed that the rotor and axle associated rapidly with an approximate association rate of 10^3^ M^−1^.s^−1^ and a Kd in the micromolar range (fig. S12). Similar results were obtained with D3-C5 rotary assemblies, and SEC and SAXS profiles were in agreement with the design model in both cases (Vr<15, Table S1–2, fig. S2, fig. S10).

**Fig. 4:**
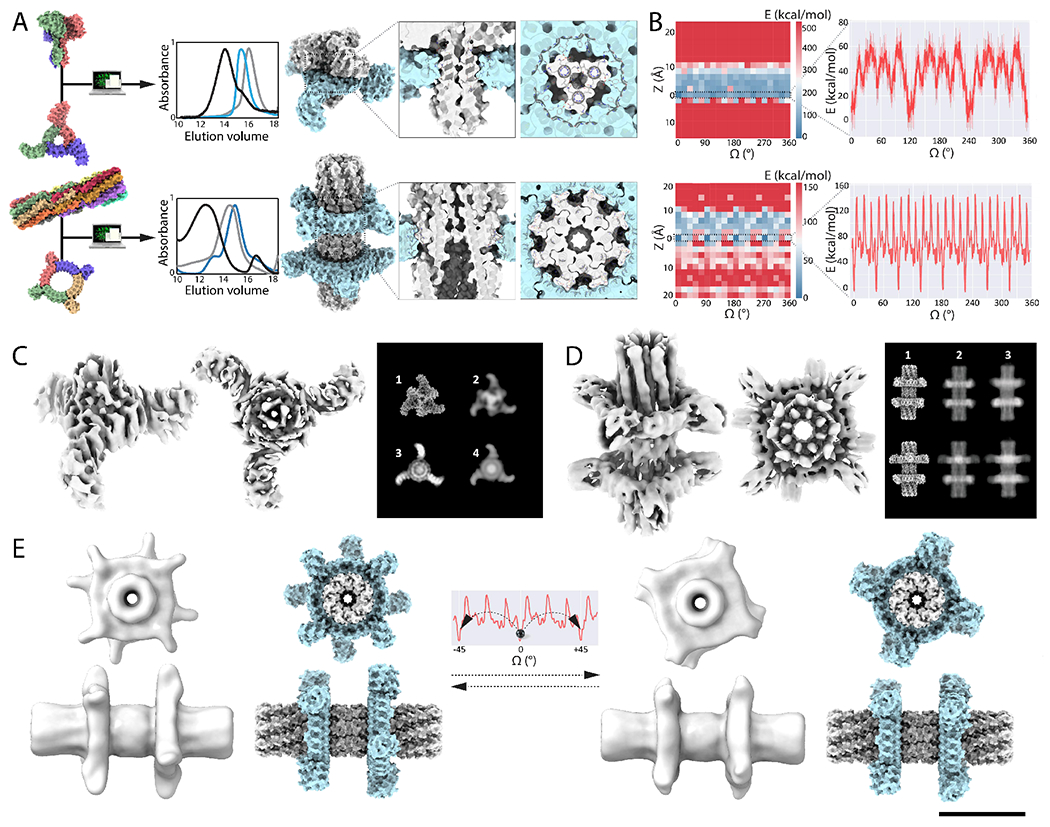
Computational sculpting of the energy landscape by design of interface side-chain interactions. (**A**) From Left to right: Models of C3 axle (C3_A1), C3 rotor (C3_R1), D8 axle (D8_1) and C4 rotor (C4_1) used to design C3-C3 and D8-C4 axle-rotor assemblies. Overlaid SEC chromatograms (absorbance at 215 nm) of axle (grey), rotor (blue), and full assembly (black). Models of symmetry matched C3-C3_1 and quasisymmetric D8-C4 assemblies and close-ups on the interface reveal the shape complementary cogwheel topology. (**B**) Energy landscapes corresponding to the C3-C3 (Top) and to the D8-C4 axle-rotor assembly (Bottom); (Left) 2D Rotation-Translation energy landscapes showing a narrow band of low energy where the rotor sits on the axle. (Right) 1D rotational energy landscape has three main minima corresponding to the C3 symmetry of the interface with 9 additional lesser energy minima for C3-C3, and eight main energy minima corresponding to the C8 symmetry of the interface and additional 18 lesser minimas for D8-C4. The energy landscapes were computed by scoring 10 independent Rosetta backbone and side-chains relax and minimization trajectories (solid red line with error bars depicting the standard deviation, kcal/mol as calculated by Rosetta) (**C**) Single particle cryoEM analysis of the C3-C3 assembly. The rotor is evident in the 6.5Å resolution electron density in the side and top views; only a portion of the axle is resolved. In the panel to the right, the experimental 2D class averages (3) match the projection of the design model more closely with conformational variability (4) than without (2); the design model is shown in (1). (**D**) Single particle cryoEM analysis of the designed D8-C4 rotor. The electron density (in grey) at 5.9Å resolution shows the main features of the designed structure and two distinct rotational states (1), also visible in the the simulated projections (2), which closely resemble the experimental 2D class average (3). (**E**) 3D variability analysis and computed rotational landscape of the D8-C4 axle-rotor assembly. The two resolved structures (shown in gray on left and right) are separated by a 45° rotational step. Corresponding computational models are shown in spacefill (blue and gray). Top row: top view, bottom row, side view. Scale bar: 10nm

Second, we experimented with more direct steric coupling to limit conformational variability primarily to rotation of the rotor around the axle. We employed shape complementary axle and rotor components to enable the incorporation of steric constraints restricting translation, leveraging Rosetta’s ability to design tightly packed interfaces and hydrogen-bond network mediated specificity(27). We designed 7 axle-rotor assemblies using this approach: three with C3 symmetric axles with C1 rotors (C3-C1_1-3, fig. S10) and four larger designs with C3 axles and rotors (C3-C3_1-4) (fig. 4A, fig. S10) with DHR arm extensions. The C3 symmetry matching of the rotor and axle differs from the mismatching in the other designed assemblies, and the extent of alignment of axle and rotor DHR arms relative to each other provides a measure of conformational variability. Design was carried out by systematically sampling rotational and translational DOF, removing arrangements with backbone to backbone clashes (see methods), and then using the Rosetta HBnet protocol and FastDesign(28) to identify interacting residues and optimize the interface energy. Each interface design trajectory generates widely different periodic energy landscapes according to interface metrics and design specifications (fig. S13), and results in shape complementary axle-rotor interfaces with an overall cogwheel topology. C3-C1 designs were experimentally screened for assembly by expressing rotor and axle pairs bicistronically and carrying out Ni-NTA purification relying on a single HIS tag on the rotor component (fig. S14A). 50% (6/12) expressed solubly and copurified, suggesting that the two components assembled in cells (fig. S14B), and three designs (C3-C1_1-3, fig. S10) were selected for further characterization. The SEC profiles in combination with native mass spectrometry indicated an oligomeric state consistent with the designed assembly, and SAXS data were also consistent with the design model (Vr<12 and MW within ~10% of expected values for C3-C1_3, and ~15% for C3-C1_1-2, Table S1–2, fig. S2, fig. S10, fig. S14C–D). The C3-C3 designs (C3-C3_1-4, fig. S10) were screened for *in vitro* assembly by stoichiometric mixing of axle and rotor, followed by SEC and SAXS analysis, which were consistent with assemblies of the expected oligomeric state (Vr<10, Table S1–2, fig. S2, fig. S10). Biolayer interferometry showed that the designed C3 axle and C3 rotor rapidly assemble with an approximate association rate of 10^3^ M^−1^.s^−1^ and a Kd in the micromolar range (fig. S12).

Third, we sought to design further constrained axle-rotor assemblies by increasing the surface area of the interfaces between axle and rotor to enable more extensive sculpting of the energy landscape. We designed a symmetry mismatched assembly consisting of a D8 axle around which two C4 rotors are assembled (D8-C4), a symmetry mismatched assembly consisting of C5 axle and C3 rotor (C5-C3_1 and C5-C3_2), as well as a C8-C4 assembly corresponding to a circular permutation version of D8-C4 (C8-C4) (fig. 4A, fig. S10). The D8-C4 assembly with one axle for two rotors tests the incorporation of multiple coupled rotational DOF in a multicomponent system and also provides a simple way to monitor the position of rotors relative to each other by experimental structural characterization. For the D8-C4, C5-C3 and C8-C4 designs, since the symmetry of the rotor is internally mismatched to the axle, we used a quasisymmetric design protocol (see methods). The C4 rotor has internally C24 symmetry, and hence is symmetry matched to both D8 and C8 axles. In contrast, the C5-C3 arrangement has broken symmetry with a resulting energy landscape with 15 energy minima, with periodicities reflecting the constituent C5 and C3 symmetries (fig. S13). Twelve D8-C4 designs, twelve C5-C3 and six C8-C4 designs were screened for *in vitro* assembly by isolating axle and rotors individually by Ni-NTA purification and mixed stoichiometrically. We selected 4 of these designs for further experimental investigation and obtained SEC data indicative of assembly of axle-rotor complexes, while SAXS analysis of a C5-C3 design suggested assembly of the axle-rotor complex (Vr=6.9 and predicted MW within 6% of expectation, Table S1–2, fig. S2, fig. S9). Biolayer interferometry binding kinetics and negative stain EM data were also consistent with quantitative assembly into the designed hetero-oligomeric complex (fig. S10, fig. S13).

## Correspondence between designed energy landscape and observed mechanical DOF

We subjected one construct from each design approach and symmetry class to single particle cryoEM examination and related these data to energy landscape calculations based on the design model (fig. 3–4). Comparison of the electron density data on the axle-rotor assemblies to data on the isolated rotors and axles suggest considerable variation in their rigid body orientations, as summarized in fig. S17–19 and S21.

For the D3-C3 and D3-C5 assemblies produced by the first approach, we obtained 2D class averages that clearly resembled projection maps computed from the design models, and 3D reconstructions in close agreement with the overall design model topology and designed hetero-oligomeric state (fig. 3C–D, fig. S15–16, Table S3). For both designs, the D3 axle was clearly visible and we obtained a high resolution structure of the axle nearly identical to the design model. 3D reconstructions in C1, C3 and D3 of the D3-C3 axle-rotor assembly at 7.8Å resolution showed clear density corresponding to the rotor in the middle of the axle with the C3 rotor arms clearly evident (fig. 3C, fig. S15). 3D reconstructions of the D3-C5 design also showed clear density for the rotor which could be isolated by masking the axle, but its resolution could not be further improved as the secondary structure placement relative to the axle appeared variable (fig. 3D, fig. S16). The particle alignment algorithm is likely dominated by features of the axle which is mostly in side-view in the data (S17–18), and thus the lack of resolution of the electron density corresponding to the rotor (see supplementary materials) is probably due to variability in the axle-rotor rigid body transform. Cryosparc 3D variability analysis(29) suggests that the rotor can populate multiple translational and rotational conformational states around the axle (Movie S1–4). Inspection of the cryoEM 3D reconstruction also suggests the rotor arms populate multiple positions along the rotational axis (fig. 3C–D, fig. S17–18). Rosetta energy landscapes generated by rotating and translating the rotor relative to the axle suggest that a broad range of orientations are energetically accessible (fig. 3B), and the rotor-axle rigid body orientation fluctuated in molecular dynamics simulations (MD), with the D3-C5 assembly showing increased displacement compared to D3-C3 (fig. 3B, fig. S11, fig. S17–18). Explicit modelling of conformational variability along the designed DOFs was necessary to produce computed projections closely resembling the experimental 2D class averages (fig. 3C–D, fig. S18). Taken together, the cryoEM data, Rosetta models and molecular dynamics simulations are consistent with the design goal of constrained mechanical coupling of axle and rotor components (see supplementary fig. S17–18 for summary of data indicating conformational sampling of rotor-axle rigid body DOFs).

Amongst the assemblies generated with the second approach, single particle cryoEM analysis of a C3-C3 assembly yielded 2D class averages with the axle and rotor clearly visible. Resolution was limited by the orientation bias of the particle in ice resulting in few side views, but we were able to obtain a 6.5Å 3D reconstruction which resembled the design model (fig. 4A, fig. S4, fig. S10, fig. S19, Table S3). 2D averages and the 3D reconstruction clearly capture the rotor component, but the axle was only partially resolved; the rotor has a mass greater than the C3 axle and clear “arm”-like features, which likely bias the alignment algorithm in its favor. Aligning on the rotor yielded a density map with diffuse density for the axle near the rotor (fig. S19). The contrast between the diffuse density for the axle and the well resolved density of the rotor likely reflects conformational variability (fig. 4C–D, fig. S4, fig. S18–19). The Rosetta energy landscape suggests that the axle-rotor assembly can primarily sample rotational rather than translational DOFs (fig. 4B), and rotational averaging increased the similarity between projections of the design model and the experimental data (fig. 4C–D, fig. S18–19). Taken together, the data (summarized in fig. S19), are consistent with variability along the rotational DOF, in accordance with the designed energy landscape which has 3 energy minima at a 60° rotation distance and 9 other 30° spaced degenerate alternative wells separated by low energy barriers (fig. 4B; fig. S13, fig. S20).

The D8-C4 design generated by the third approach has a rugged energy landscape, with a dynamic range of 151 kcal/mol (as estimated by Rosetta), with 8 steep wells spaced 45° stepwise along the rotational axis corresponding to the high symmetry of the interface (fig. 4B). Consistent with the deep minima in this landscape, we obtained a cryoEM map of ~5.9Å resolution that is close to the design model (fig. 4C–D, fig. S6, Table S3). 3D variability analysis calculations using Cryosparc(30) suggested two nearly equiprobable states in which the rotor arms are either aligned or offset, as in the eclipsed and staggered arrangements of ethane (fig. 4D–E, fig. S6, fig. S18, fig. S21, Movie S5). The two rotational states of one rotor relative to the other suggest energy minima spaced by 45° along the rotational axis, consistent with an 8-fold step like feature in the frequency spectrum analysis of the computed energy landscape (fig. S21). While cryoEM provides a frozen snapshot of molecules and not a real time measurement of diffusion, these data (summarized in fig. S20) suggest that the system populates multiple rotational states consistent with the designed energy landscape. Taken together, these results suggest that the explicit design of side-chain interactions and deep energy minima reduces the degeneracy of conformational states observed with purely electrostatic interactions, and support a correspondence between the energy landscape and the observed conformational variability.

## Conclusions

Our proof of concept axle-rotor assemblies demonstrate that protein nanostructures with internal mechanical constraints can now be systematically designed. Key to this advance is the ability to computationally design protein components with complex complementary shapes, symmetries and topologies, such as the high aspect ratio dihedral axle structures (D2 homotetramers to D8 homo-16-mers (fig. 1–2) with oligomerization states and sizes considerably larger than previously designed dihedral structures. Our studies of assembly of these shape complementary homo-oligomeric components into higher order hetero-oligomeric structures with internal degrees of freedom provide insights towards the design of complex protein nanomachines. First, computational sculpting of the interface between the components can be used to promote self-assembly of constrained systems with chosen internal degrees of freedom. Second, the shape and periodicity of the resulting energy landscape is determined by the symmetry of components, the shape complementarity of the interface, and the balance between hydrophobic packing and conformationally promiscuous electrostatic interactions (fig. 3A–B, fig. 4A–B). Symmetry mismatch generates assemblies with larger numbers of energy minima than symmetry matched ones evident in the frequency domain (fig. S13, fig. S20), and explicit design of close sidechain packing across the interface results in deeper minima and higher barriers than non-specific interactions (fig. 3–4, fig. S13). In general, the surface area of the interface between axle and rotor scales with the number of subunits in the symmetry, with larger surface areas providing a larger energetic dynamic range accessible for design (fig. 3–4, fig. S13). The combination of the conformational variability apparent in the cryoEM data of D3-C3, D3-C5 and C3-C3 designs (fig. 3C–D, fig. 4C–D, fig. S4, fig. S15–19), the Rosetta and MD simulations (fig. 3B, fig. 4B, fig. S11), and the discrete states observed for the D8-C4 design (fig. 4D–E, fig. S6, fig. S21), suggests that these assemblies populate multiple rotational states (the axle-rotor assemblies also have multiple symmetrically identical yet physically distinct rotational states—for example, rotation of the C3 rotor around the C3 axle by 120 degrees--which cannot be distinguished by cryoEM). Our cryoEM analysis cannot distinguish whether the conformational variability reflects rotational motion or states captured during axle-rotor assembly, and do not report on energy barrier heights; time-resolved microscopy at the single molecule level will be required to reveal the dynamics of transitions between the different states, and relate the computational sculpting of the rotational energy landscapes to Brownian dynamics.

The internal periodic but asymmetric rotational energy landscapes of our mechanically coupled axle-rotor systems provide one of two needed elements for a directional motor. Coupling to an energy input to break detailed balance and drive directional motion remains to be designed: for example the interface between machine components could be designed for binding and catalysis of a small molecule fuel (22). Symmetry mismatch, which plays a crucial role in torque generation in natural motors (31,32), can incorporated synthetic protein motors as illustrated here for our rotor-axle assemblies. Modular assembly could lead to compound machines for advanced operation or integration within nanomaterials, and the components can be further functionalized using reversible heterodimer extensions (34) (fig. S22). Our protein systems can be genetically encoded for multicomponent self-assembly within cells (fig. S14) or *in vitro* (fig. S9, fig. S12) Taken together, these approaches could enable the engineering of a range of nanodevices for medicine, material sciences or industrial bioprocesses. More fundamentally, *de novo* design provides a bottom-up platform to explore the fundamental principles and mechanisms underlying nanomachine function that complements long standing studies of the elaborate molecular machines produced by natural evolution.

## Supplementary Material

Supplementary_Information

figS1

Movie_S4

Movie_S5

Movie_S2

Movie_S3

Movie_S1

Data S3

Data S2

Data S1

Data S4

Data S5

## Data Availability

All data are available in the main text or the supplementary materials. All the EM maps have been deposited in the EMDB (accession codes EMD-25575, EMD-25576, EMD-25577, EMD-25578, EMD-25579, EMD-25580).
