# Supplementary material for: Computational design of mechanically coupled axle-rotor protein assemblies
Source: Science. Author manuscript; Available in PMC 2023 Dec 11. (PMC10712554; doi:10.1126/science.abm1183)
Supplement: Supplementary_Information [file NIHMS1871500-supplement-Supplementary_Information.pdf]

Science

AAAS

Supplementary Materials for

Computational design of mechanically coupled axle-rotor protein assemblies

A. Courbet[1,2,3,†], J. Hansen[1,†], Y. Hsia[1,2], N. Bethel[1,2], YJ. Park[1], C. Xu[1,2], A. Moyer[1,2], S.E. Boyken[1,2,‡], G. Ueda[1,2], U. Nattermann[1,2], D. Nagarajan[1,2], D. Silva[1,2], W. Sheffler[1,2], J. Quispe[1], A. Nord[4], N. King[1,2], P. Bradley[5], D. Veesler[1], J. Kollman[1], D. Baker[1,2,3]*

Correspondence to: dabaker@uw.edu

This PDF file includes:

Materials and Methods

Figs. S1 to S22

Tables S1 to S3

Captions for Movies S1 to S5

Other Supplementary Materials for this manuscript include the following:

Movies S1 to S5

Data S1 to S5

Materials and Methods

Computational Design

Generation of homooligomeric dihedral and cyclic symmetric axle parts:

Approach 1: This approach relies first on the design of short (30 to 50 residues) single alpha helices monomers self-assembling into high aspect ratio dihedral homoligomers, which are then further fused to cyclic wheel shaped homooligomers to yield full axle parts.

Parametric design was used to generate short single α-helices and sample backbone configurations by systematically varying helical parameters using the Crick generating equations(35,*36*). As described before, ideal values were used for the supercoil twist (ω0) and helical twist (ω1).

In the case of D3 helical bundles, in order to obtain the helical interdigitated geometry allowing the obtain a packed core without holes after design and therefore assembly of single helices into dihedral symmetry, we sampled two segments with different starting point for the superhelical radii per helix (6Å and 12Å), joined by a custom number of linker residues between the two segments, using a custom python script. This parameter range (~5Å with bins of 0.5Å) were chosen based on iteratives cycles of parametric helix generation and Rosetta design with metric assessment, and the range of metrics yielding the highest scoring backbone were chosen. The helical phase (Δϕ1) was sampled from 0° to 90° with a step size of 10°. We sampled the offset along the z-axis (Z-offset) from -1.51Å to 1.51Å, with a step size of 0.1Å. The supercoil phases (Δϕ0) were fixed at 0°, and 30° for D3s and D2s, respectively.

Once ideal backbones geometry were generated using this parametric approach, we used the Rosetta design protocol to further design side chains identities and rotamers and optimize the interface energy to direct the assembly in dihedral homooligomers. Importantly, this step relied on the use of the Rosetta HBnet protocol described previously(*27*), which allows for extended hydrogen bond networks across monomer subunits therefore ensuring specificity of interaction and symmetric binding mode.

The dihedral building blocks were then rigidly fused to previously designed cyclic homooligomers(*36*) by designing short rigid helical linkers bridging the two building blocks. The inner helices of the dihedral assemblies obtained (C or N termini depending on design) were then fused by short structured helical fragments using Rosetta Remodel(37) while sampling the rotation and distance between Z aligned cyclic homooligomers and dihedral homooligomer. To further stabilize and optimize the generated Cyclic-Dihedral fusion, a second round of Rosetta design of the fusion was performed.

Method 2: This approach relied on alpha helical extensions of N or C termini of previously designed cyclic homooligomers, in order to direct the assembly of two elongated cyclic homooligomers into high aspect ratio dihedral symmetric axle parts. Rosetta SymDofMover was

used to set up the symmetry in which the input monomer subunits were aligned along the z axis. Input subunits were first optionally flipped 180 degrees about the z axis to reverse the inputs if necessary, so that the N or C termini to be elongated would point toward each other. Monomer subunits were then translated along the specified z axis and rotated about the z axis according to random Gaussian sampling in order to finely sample helical extension parameters. Following these initial manipulations of the input structures, a symmetric pose was generated using D3, D4, D5, D6 or D8 symmetry definition files. We then applied the Rosetta BluePrintBDR mover which allowed us to build helical fragment extension starting at the previously positioned monomers, and spanning the distance between symmetric subunits. Once centroid helical backbones geometries were generated and sampled, we used the Rosetta design protocol to further design side chains identities and rotamers and optimize the interface energy to direct the assembly in dihedral homooligomers. Importantly, this step relied on the use of the Rosetta HBnet protocol described previously(*27*), which allows for extended hydrogen bond networks across monomer subunits therefore ensuring specificity of interaction and symmetric binding mode.

<u>Generation of cyclic symmetric homooligomeric rotor parts:</u>
Computationally designed ring shape structures or various symmetries (C1, C3, C4), were either collected from previously published work(*24,36*), or designed from heterodimers and DHRs in symmetry mode (C3, C5) using protocols previously described(*14*). 9x, 12x and 24x toroids were used in C1 symmetric versions or cut into 3 or 4 to produce C3 or C4 symmetric homooligomers. All designs were then computationally augmented by systematic symmetric fusion of DHR repeats proteins using the HFuse protocol, and the surrounding fusion interface of the fusion was further redesigned using Rosetta design protocols to optimize the assembly energy.

<u>Generation of two component rotary machine models from symmetric axle and rotor parts:</u>
The goal of the computational docking procedure between axle and rotor machine parts was to exhaustively sample the rotational conformational space within some specified resolution and meaningful interface quality, all possible ways to assemble a full rotary machine complex from the two libraries of previously designed axle and rotor parts.

We started by enumerating all possible rotary machine assemblies by inspecting shapes and dimensions of available parts and identifying assemblies that would not produce any steric clashes. We then proceeded to computational docking of parts using a two-dimensional rigid body docking space to allow contact between the axle and rotor (one rotation and one translation along the Z axis). We sampled 180° rotation for C2s, 120° for C3s, 90° for C4s, 72° for C5s, and we sampled the whole span on available translation along the axis that would not generate clashes between backbones, with a 1° and 1A step, respectively. For each sampled dock, the resulting heteromultimeric interface was designed either using Rosetta design and HBnet to obtain tightly packed, specific interfaces with extended hydrogen bond networks, and in some cases by constraining the residue identities of the axle (DEHQTNSY) and rotor (KRHQTNSY) to obtain complementary charges allowing loose non specific interactions. Since some of the resulting assemblies have intrinsic symmetry mismatch between the axle and rotor (*e.g.* D8 axle and C4 rotor), we used a quasi-symmetric design methodology, relying on the Rosetta StoreQuasiSymmetricTaskMover, which creates a stored task that links selected interface residues. The residues remain identical in identity when the interface is designed, but their

rotamers are packed differently, which allows identical residues in symmetric subunits to satisfy multiple interfaces at the same time.

Interface disulfide stapling

In order to kinetically trap rotors onto the axle, we further generated a disulfided version of homooligomers by placing cysteine at the interface between asymmetric units. This was achieved using a PyRosetta based stapling method that allows to identify pairs of residues that can accommodate disulfides given the 3D structure of a protein. This protocol was developed to quickly identify pairs of residues that can accommodate disulfides given the 3D structure of a protein. 30,000 native disulfide structures were procured from the PDB, and the relative positions of the backbone atoms (N, CA, C) were calculated, hashed, and stored into a database. A candidate protein structure can then be searched for residue pairs at all relative positions of backbone atoms that can accommodate disulfides according to native geometries. (https://github.com/atom-moyer/stapler)

Molecular dynamics simulations

Rosetta models of D3-C3 and D3-C5 with truncated rotor DHR arms (to minimize the total number of atoms to simulate) were used as the starting coordinates for the simulations. The rotor rotors of D3-C3 and D3-C5 were rotated at 10 and 12 degree intervals, respectively. Each model was solvated in an octahedral periodic box of OPC water and 70 mM NaCl using AmberTools18(39). In total, each system consisted of approximately 590,000 atoms. Simulations were run at constant pressure (1 bar) and temperature (298 K) using the Monte Carlo barostat, the Langevin thermostat and the ff19SB forcefield(*40*). Using the CUDA enabled version of Amber18, four parallel simulations for each rotated model were equilibrated using the AmberMDprep protocol(*41*). Once equilibrated, the simulations were run at 2 fs timestep for a total of 40 ns each, yielding an aggregate simulation time of 1920 ns for D3-C3 and 960 ns for D3-C5. To allow exploration of the rotors' degrees of freedom from the initial configurations, the first 20 ns of each simulation was discarded and the final 20 ns was used in later analysis. To investigate the movement of the rotors around their respective axles, 200 ps snapshots of the simulations were aligned to the initial axle coordinates by RMSD. Number density maps of the backbone atoms were calculated using the VolMap command in AmberTool's cpptraj(*42*). These maps were contoured to 0.001 as shown in **fig. S10**. To calculate the axle drift with respect to rotor rotation, the backbone center of mass of the rotors was calculated for all aligned snapshots. The snapshots were binned according to the rotor rotation in 24 degree intervals and then averaged as shown in **fig. S10**. To calculate rotor tilt, the centers of mass of each rotor subunit was calculated, then a plane was fit through these points using the least squares optimizer in SciPy(*43*). The angle between this plane and the long axis of the axle was taken as the tilt, and this was averaged over rotation as described for the axial drift. The Mean Square Displacement for the DOFs was computed as MSD=average(r(t)-r(0))^2.

All PDBs can be found in DataS5. All scripts and computational methods are accessible on GitHub (https://github.com/alexiscourbet)

Buffer and media recipe for protein expression

TBM-5052: 1.5% [wt/vol] tryptone, 2.5% [wt/vol] yeast extract, 0.5% [wt/vol] glycerol, 0.05% [wt/vol] D-glucose, 0.2% [wt/vol] D-lactose, 25 mM Na2HPO4, 25 mM KH2PO4, 50 mM NH4Cl, 5 mM Na2SO4, 2 mM MgSO4, 10 μM FeCl3, 4 μM CaCl2, 2 μM MnCl2, 2 μM ZnSO4, 400 nM CoCl2, 400 nM NiCl2, 400 nM CuCl2, 400 nM Na2MoO4, 400 nM Na2SeO3, 400 nM H3BO3

Lysis buffer: 25 mM Tris, 25 mM NaCl, 20 mM Imidazole, pH 8.0 at room temperature

Wash buffer: 25 mM Tris, 25mM NaCl, 20 mM Imidazole, pH 8.0 at room temperature

Elution buffer: 25 mM Tris, 25 mM NaCl, 200 mM Imidazole, 50mM EDTA, pH 8.0 at room temperature

TBS buffer: 25 mM Tris pH 8.0, 25 mM NaCl

Construction of synthetic genes

Prior to transformation and expression in E coli hosts, synthetic genes were ordered either from Integrated DNA Technologies (Coralville, IA) or Genscript Inc. (Piscataway, N.J., USA) and cloned in pET29b+ e. coli expression vector between the NdeI and XhoI sites. For bicistronic constructs used for screening the *in cellulo* assembly of axle and rotors, a synthetic bicistron containing both axle and rotor genes were synthesised and cloned at once in the Nde1/XhoI site, with a termination and strong ribosomal binding site sequence between the genes. For most synthetic gene constructs, a C or N ter hexahistidine tag was added in frame after a short GS linker. A stop codon was introduced at the 3' end of the protein coding sequence to prevent expression of the C-terminal hexahistidine tag in the vector.
(See Data_S4_components_and_machines_sequences.fasta for all sequences)

Protein expression

Plasmids were transformed into chemically competent E. coli expression strain BL21(DE3*) (New England Biolabs) for protein expression. Following transformation and overnight growth on Luria-Bertani agar Kanamycin plates 100ug/ml, single colonies were picked and directly transferred into 2x50 ml TBM-5052 medium containing 150 μg/mL Kanamycin and incubated with shaking at 225 rpm for 24 hours at 37°C following the autoinduction method(*44*). After 24 hours of incubation, the temperature was dropped for an overnight incubation at 20°C before harvesting the cells via centrifugation at 4500G for 20 minutes at 4°C.

Affinity purification

The cell pellets were resuspended in 30ml lysis buffer, followed by cell lysis via sonication at 85% power for 2.5 minutes (10 sec on/10 sec off) while keeping the cell suspension at 4°C. Lysates were clarified by centrifugation at 4°C and 18000 G for 45 minutes and applied to columns containing Ni-NTA (Qiagen) resin pre-equilibrated with lysis buffer. The columns were

washed 3 times with 10 column volumes (CV) of wash buffer, followed by 15ml of elution buffer for protein elution.

Size-exclusion chromatography (SEC)

Protein elutions were further concentrated in 15mL 3K protein concentrators (Millipore Sigma) to a volume of 500uL and the buffer exchanged for TBS buffer. The resulting protein solutions were purified by SEC using a Superdex 6 10/300 GL increase column (GE Healthcare) or a Superdex 200 10/300 GL increase column in TBS buffer. SEC elution fractions corresponding to the designs theoretical elution volumes were concentrated in TBS prior to further biochemical analysis. The experimental elution volumes were calculated by using curve fitting available in Unicorn 7.3 software (GE Healthcare). The predicted SEC elution volumes were computed from PDB models using the following equations, which were obtained via regression using a large number of various *de novo* proteins of know masses and recording their elution volumes with the same buffer and column (R^2=0.975 and R^2=0.971 for S200 and S6, respectively): $V_{S200} = -1.89log(<mass\ of\ design\ in\ kDa>) + 21.9$; and $V_{S6} = -1.33log(<mass\ of\ design\ in\ kDa>) + 21.9$. Figure S2A and Table S1 detail the analysis of SEC data compared with the prediction from models for the whole library of computationally designed protein components. Predicted elution profiles are highly correlated with the experimental elution profiles for the whole library of designs. All SEC traces can be found in Data S3.

D3-C3 and D3-C5 assembly process

D3 axles and C3 or C5 rotors were purified as previously described. Axle and rotor were then mixed in TBS solution with 25mM TCEP following a 1:1 stoichiometry, after which the pH is dropped to 3.0 by dialysis in citrate buffer with TCEP. The protein samples were then heated for an hour at 65C, and then allowed to cool back down to room temperature on a bench. The protein samples were then dialysed overnight in TBS buffer and further SEC purified.

Small Angle X-ray Scattering (SAXS)

Protein samples were purified by SEC in 25 mM Tris pH 8.0, 25 mM NaCl and 1% glycerol; elution fractions corresponding to the protein were further concentrated using 3K protein concentrators (Millipore Sigma) and the flow-through was used as blank for buffer subtraction. SAXS Scattering measurements were performed at the SIBYLS 12.3.1 beamline at the Advanced Light Source. The sample-to-detector distance was 1.5 m, and the X-ray wavelength (λ) was 1.27 Å, corresponding to a scattering vector q ($q = 4\pi \sin \theta/\lambda$, where 2θ is the scattering angle) range of 0.01 to 0.3 Å^{-1}. A series of exposures were taken of each well, in equal sub-second time slices: 0.3-s exposures for 10 s resulting in 32 frames per sample. For each sample, data were collected for two different concentrations to test for concentration-dependent effects; 'low' concentration samples corresponded to 1 mg/ml and 'high' concentration samples to 5 mg/ml or the highest protein concentration achievable. Collected data were processed using the SAXS FrameSlice online server and analysed using the ScÅtter software package(*45*). The FoXS software (Sali Lab), and SAXSMoW were used to compare experimental scattering profiles to design models and assess quality of fit by computing Volatility ratios (Vr), $\chi2$, Radius of gyration (Rg) and predicted molecular weight (25,*46,47*). We decided to use both $\chi2$ and Vr

because Vr was previously shown to be a superior metric for comparing SAXS profiles (26), as it measures differences of normalized SAXS curves across the entire resolution range of scattering vectors and assesses structural similarity objectively. Vr is more sensitive to differences at high and low q-values which allows better assessment of the agreement between experimental scattering data and design models. Assemblies that had SEC elution profiles and EM maps consistent with the design models, and SAXSMOW radius of gyration and molecular weight within 10% of the design model, had Vr values between scattering profile and design model of less than 13, and for assemblies where we have less extensive data we consider profiles with Vr<15 to be consistent with the design model. Overall the SAXSMOW molecular weights and radii of gyration derived from the SAXS traces of the axle, rotor and axle-rotor assemblies are strongly correlated with those derived from the computational models although there are deviations in individual cases (Figure S2B-C and Table S2). All SAXS traces can be found in Data S2.

Electron microscopy

Negative Stain Electron Microscopy:

SEC fractions corresponding to the designs were concentrated in TBS prior to negative stain EM screening. Samples were then immediately diluted 5 to 150 times in TBS buffer (tris 25mM, NaCl 25mM) depending on the concentration of the samples. A final volume of 5 µL was applied on negatively glow discharged, carbon-coated 400-mesh copper grids (01844-F, TedPella,Inc.), then washed with Milli-Q Water and stained using 0.75% uranyl formate as previously described(*48*). Air-dried grids were then imaged on either a FEI Talos L120C TEM (FEI Thermo Scientific, Hillsboro, OR) equipped with a 4K × 4K Gatan OneView camera at a magnification of 57,000x and pixel size of 2.51Å. Micrographs collection was automated using EPU software (FEI Thermo Scientific, Hillsboro, OR) and were imported into CisTEM software(49) or cryoSPARC software(29-30). CTF estimation was done with CTFFIND4 and a circular blob picker was used to select particles which were then subjected to 2D classification. Ab initio reconstruction and homogeneous refinement in Cn symmetry were used to generate 3D electron density maps.

CryoEM Sample Preparation and DataCollection:

CryoEM grids were prepared by diluting protein samples with TBS 1 to 10 times immediately before applying 3.5 µL to glow-discharged 400 mesh, C-flat, 2 micron holes, 2 micron spacing, CF-2/2-4C (CF-224C-100) (Electron Microscopy Sciences, Hatfield, PA) cryoEM grids. For some samples, multiple blots were applied in order to obtain the best particle density. All grids were blotted using a blot force of 0 and 5 second blot time at 100% humidity and 4°C and plunge-frozen in liquid ethane using a Vitrobot Mark IV (FEI Thermo Scientific, Hillsboro, OR). All cryoEM grids were screened on a Glacios transmission electron microscope (FEI Thermo Scientific, Hillsboro, OR) operated at 200 kV and equipped with a Gatan K2 Summit direct detector. Automated glacios data collection was carried out using Leginon(*50*) at a nominal magnification of 36,000x (1.16 Å/pixel). Movies were acquired in counting mode fractionated in 50 frames of 200 ms at 8.5 e-/pixel/sec for a total dose of ~65e-/Å^2. High resolution data was collected on a Titan Krios (FEIco.) operating at 300kV, with a Quantum GIF energy filter (GatanInc.) operating in zero-loss mode with a 20eV slit width, and a K-2 Summit Direct Detect camera. Movies were acquired using Leginon in super-resolution mode at 130,000X (pixel size

0.525Å/pixel) with 50 frames at an exposure rate of 2.5 e-/pixel/sec for a total dose of ~90e/Å^2. Details of data processing for each design are illustrated in Table S3 and Figure S4-7, S15 and S16. Theoretical 2D projections were generated using CryoSparc software's "create template" function from an input volume generated with EMAN2(*51*).

CryoEM data processing:

Multiple datasets were collected for each design and combined early on during processing. See table 1 and processing flowcharts for details. Briefly, images were manually curated to remove poor quality acquisitions such as bad ice or large regions of carbon. Dose-weighting and image alignment of all 50 frames was carried out using MotionCor2(52) with 5X5 patch or with cryosparc v2 patch alignment tool with default parameters. Super-resolution krios data was binned 2X during alignment. Initial CTF parameters were estimated using CTFfind4(53). Particle picking was done with a gaussian blob picker and in some cases followed by a template picker. Particles were extensively classified in 2D to remove junk particles and designs which may not have been intact or were damaged, yielding in some cases relatively few particles. This may also be due to the low mass of the designed proteins which did not align well. In addition, the expected motion of the rotors may have introduced further heterogeneity, limiting classification efforts. Starting models for all designs were always obtained *ab initio*, despite clear evidence of the expected design in 2D. In 3D classification and refinement we were able to resolve either axle or rotor, and in one case both together (D8-C4), suggesting rotor movement. FSC 0.143 curves were generated by exporting half maps to relion for post-process. Local resolution estimates were generated in relion and displayed onto the locally filtered map outputs using Chimera(54). For density modification (Phenix(55)) we used as input the exported half maps from cryosparc with default params at 100 bins and local filtering with a factor of 5. FSC curves were plotted using the Phenix density modification Fref 0.5 output along with the relion FSC estimates. Directional FSC calculated using remote 3DFSC processing tool (https://3dfsc.salk.edu/) and displayed as FSC volume as previously described (56). 3D Variability analysis(30) (3DVA) was used as an alternative to 3D classification to identify distinct states in our data. Briefly, a consensus map is provided along with the particles and their alignments which were used to generate the reconstruction. Per-particle variability is calculated in terms of principal components in the user-defined number of modes to capture variability within the data. The results are smoothed and interpolated to create a user-defined number of discrete states. This method is used for identifying flexibility, motion, and discrete heterogeneity within proteins. 3DVA of the D8-C4 design was processed in cryosparc v2 following particle expansion in D4 symmetry of the final reconstructions with a mask around both rotors and the axel. We used default settings of simple cluster mode and 10 frame output with a 10Å lowpass filter for assessing variability. First and last frames of the second trajectory component were used as input for heterogeneous refinement (classification) of the particles which initially went into the 3DVA, yielding two classes with approximately equal number of particles. Resulting maps were then low-pass filtered to 15Å for clarity. For D3-C5 and D3-C3, 3DVA was carried out after D3 symmetry was expanded and variability was processed and filtered at 5Å for display. We used defaults settings for simple cluster mode and 20 frame output with 5Å filter to assess variability of three principle components. All 20 frames were used in the generation of movies. All the EM maps have been deposited in the EMDB (accession codes: EMD-25575, EMD-25576, EMD-25577, EMD-25578, EMD-25579, EMD-25580).

Biolayer interferometry

Biolayer interferometry experiments were performed on an OctetRED96 BLI system (ForteBio, Menlo Park, CA). Enzymatic protein biotinylation was performed on SEC purified Avi-tagged proteins prior to the assay. The BirA500 (Avidity, LLC) biotinylation kit was used to biotinylate protein from the IMAC elution according to the manufacturer protocol. Reactions were incubated at 4C overnight and purified using size exclusion chromatography on a Superdex 6 10/300 Increase GL (GE Healthcare) in TBS buffer (25 mM Tris pH 8.0, 25 mM NaCl). Streptavidin coated biosensors were equilibrated for 10 minutes in Octet buffer (10 mM HEPES pH 7.4, 25 mM NaCl, 3 mM EDTA, 0.05% Surfactant P20) supplemented with 1 mg/ml Bovine Serum Albumin (SigmaAldrich). Enzymatically biotinylated axle components were immobilized onto the biosensors by dipping the biosensors into a solution with 10-50 nM protein for 200-500s. This was followed by dipping in fresh octet buffer to establish a baseline. Titration experiments were performed at 25 °C while rotating at 1,000 r.p.m. Association of rotors rotor components with axle immobilized on the tips was allowed by dipping biosensors in solutions containing designed protein diluted in octet buffer followed by dissociation by dipping the biosensors into fresh buffer solution in order to monitor the dissociation kinetics. The experimental data was fitted using the ForteBio analysis software to obtain k_{obs} and k_{diss} parameters, which were then used to calculate the association rates ($Ka = k_{obs} - k_{diss}$ / [Analyte]).

Native Mass Spectrometry

The oligomeric state of *in vivo* assembled rotors was analyzed by online buffer exchange MS(57) using a Vanquish UHPLC coupled to a Q Exactive Ultra-High Mass Range (UHMR) mass spectrometer (Thermo Fisher Scientific) modified to allow for surface-induced dissociation (SID) similar to that previously described(58). 1 µL of 25 µM protein in TBS buffer were injected and online buffer exchanged into 200 mM ammonium acetate, pH 6.8 by a self-packed buffer exchange column (P6 polyacrylamide gel, Bio-Rad Laboratories) at a flow rate of 100 µL per min. A heated electrospray ionization (HESI) source with a spray voltage of 4 kV was used for ionization. Mass spectra were recorded for 1000 –20000 m/z at 3125 resolution as defined at 400 m/z. The injection time was set to 200 ms. Voltages applied to the transfer optics were optimized to allow for ion transmission while minimizing unintentional ion activation, and a higher-energy collisional dissociation of 5 V was applied. Mass spectra were deconvolved using UniDec V4.2.2 22. Deconvolution settings included mass sampling every 10 Da, smooth charge states distributions, automatic peak width tool, point smooth width of 1 or 10, and beta of 50.

Visualization and figures

All structural images for figures were generated using PyMOL, Chimera or ChimeraX. Data was processed and figures were plotted using Pandas, MatplotLib, and Seaborn python libraries. Figures were further rendered and assembled using Adobe Illustrator.

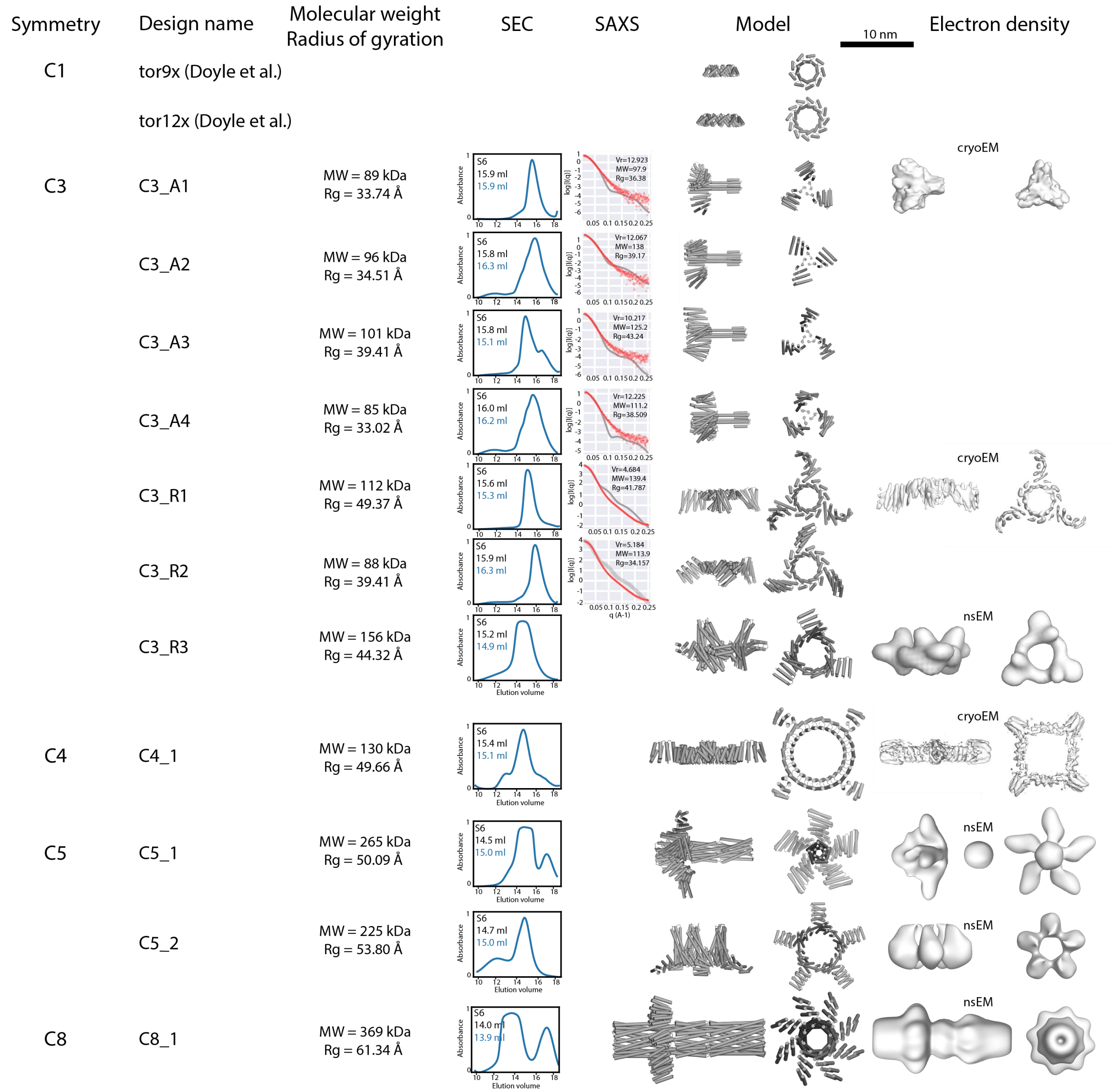

Figure S1: Detail of the library of axle and rotor parts for the design of protein machines with corresponding symmetry, design nomenclature, oligomeric mass (MW) and radius of gyration (Rg) of model, SEC chromatograms, SAXS traces, PDB model, and 3D electron density reconstruction from electron microscopy analysis. For each SEC trace, the predicted elution volume corresponding to the correct oligomer state is given in milliliters in black next to the chromatogram, and the experimental elution volume obtained by fitting the peak is reported in blue (All reported in Table S1). Experimental SAXS traces are shown in the red line, while the theoretical trace corresponding to the design is shown in grey. Volatility ratios (Vr), predicted molecular weight (MW) and radius of gyration (Rg) were computed and shown with each trace, and reported in Table S2. nsEM: data obtained using negative stain electron microscopy processed in symmetry mode; cryoEM: data obtained using single particle cryoelectron microscopy processed in symmetry mode.

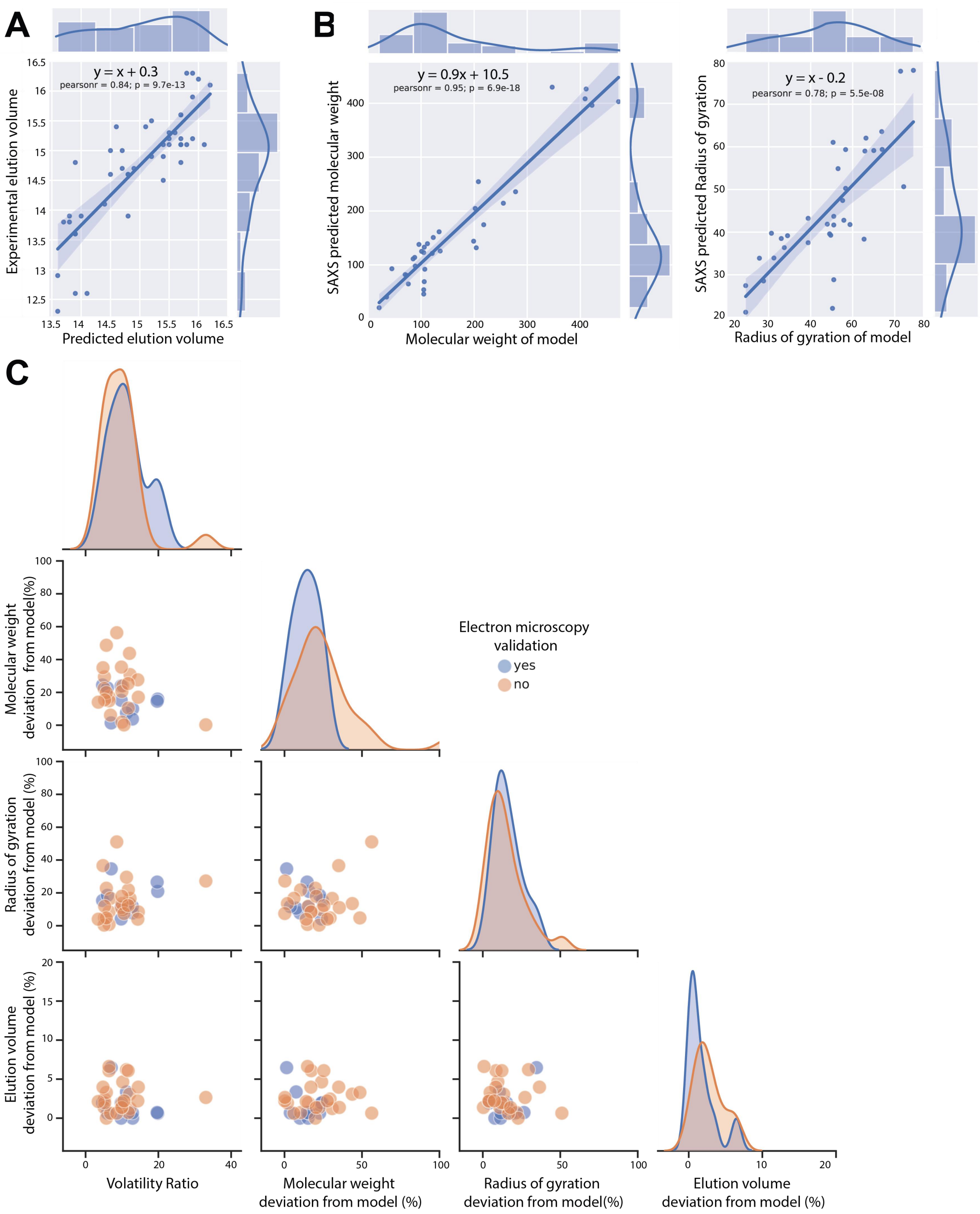

Figure S2: Detailed analysis of SEC and SAXS data compared with the prediction from models for the library of computationally designed protein components. (**A**) Detail of comparison between the size exclusion chromatography experimental elution volumes versus the predicted elution volumes. Predicted elution profiles are highly correlated with the experimental elution profiles. (**B**) Detail of comparison between the small angle X-ray scattering metrics computed from the experimental data and the metric computed from the PDB models, molecular weight (left) and radius of gyration (right). SAXS predicted molecular weights and radii of gyration are highly correlated with those computed from PDB models. (**C**) Overview and pairwise comparison between the metrics computed from the small angle X-ray scattering and SEC data over the library of designed protein components. Blue dots correspond to design that were structurally validated by electron microscopy. Assemblies that had SEC elution profiles and EM maps consistent with the design models, and SAXSMOW radius of gyration and molecular weight within 10% of the design model, had Vr values between scattering profile and design model of

less than 13, and for assemblies where we have less extensive data we consider profiles with Vr < 15 to be consistent with the design model. Overall the SAXSMOW molecular weights and radii of gyration derived from the SAXS traces of the axle, rotor and axle-rotor assemblies are strongly correlated with those derived from the computational models although there are deviations in individual cases (Figure S2B-C and Table S2). All SAXS traces can be found in Data S2.

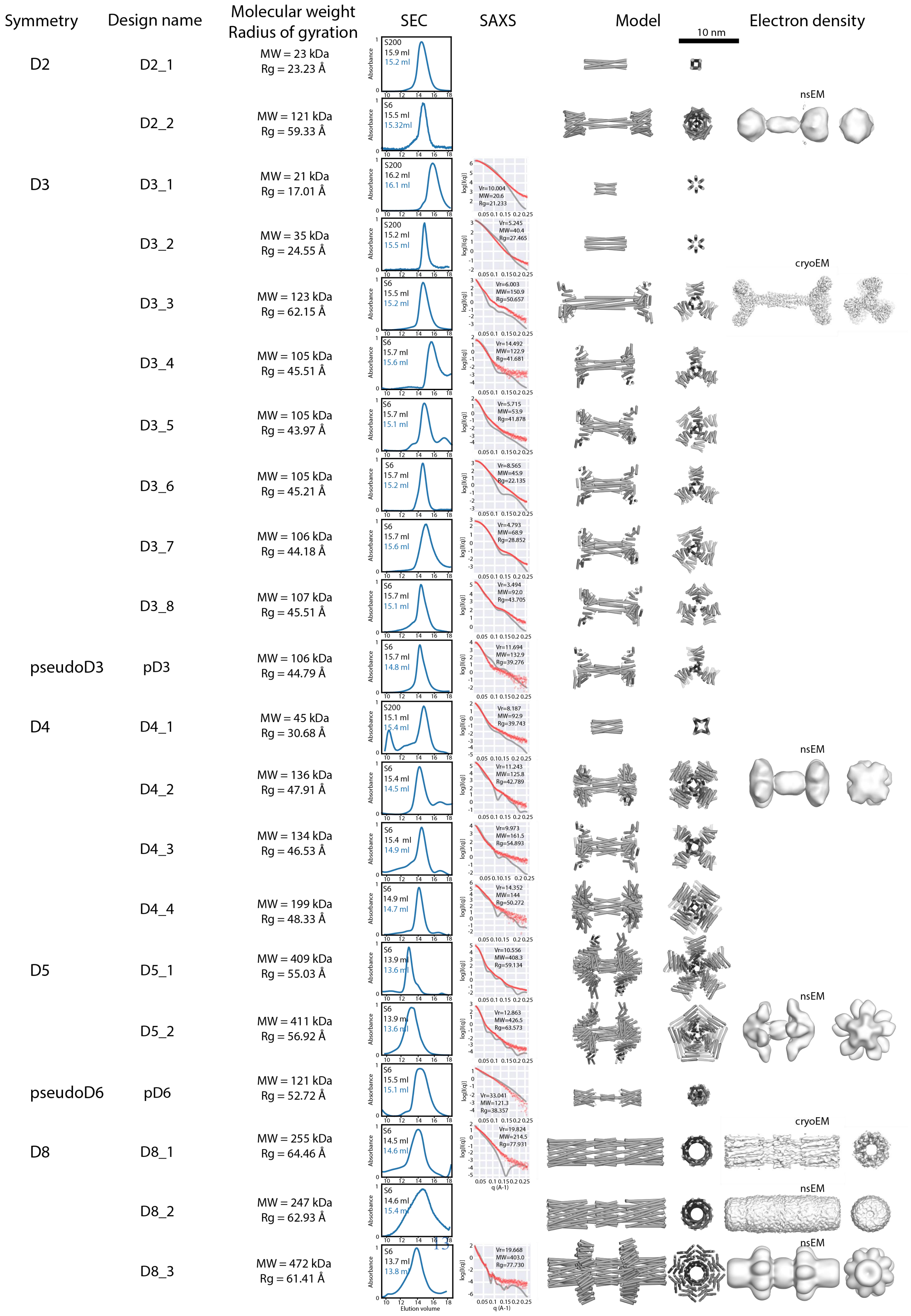
Symmetry
Design name
Molecular weight
Radius of gyration
SEC
SAXS
Model
10 nm
Electron density
D2
D2_1
MW = 23 kDa
Rg = 23.23 Å
S200
15.9 ml
15.2 ml
D2_2
MW = 121 kDa
Rg = 59.33 Å
S6
15.5 ml
15.32ml
nsEM
D3
D3_1
MW = 21 kDa
Rg = 17.01 Å
S200
16.2 ml
16.1 ml
Vr=10.004
MW=20.6
Rg=21.233
D3_2
MW = 35 kDa
Rg = 24.55 Å
S200
15.2 ml
15.5 ml
Vr=5.245
MW=40.4
Rg=27.465
cryoEM
D3_3
MW = 123 kDa
Rg = 62.15 Å
S6
15.5 ml
15.2 ml
Vr=6.003
MW=150.9
Rg=50.657
D3_4
MW = 105 kDa
Rg = 45.51 Å
S6
15.7 ml
15.6 ml
Vr=14.492
MW=122.9
Rg=41.681
D3_5
MW = 105 kDa
Rg = 43.97 Å
S6
15.7 ml
15.1 ml
Vr=5.715
MW=53.9
Rg=41.878
D3_6
MW = 105 kDa
Rg = 45.21 Å
S6
15.7 ml
15.2 ml
Vr=8.565
MW=45.9
Rg=22.135
D3_7
MW = 106 kDa
Rg = 44.18 Å
S6
15.7 ml
15.6 ml
Vr=4.793
MW=68.9
Rg=28.852
D3_8
MW = 107 kDa
Rg = 45.51 Å
S6
15.7 ml
15.1 ml
Vr=3.494
MW=92.0
Rg=43.705
pseudoD3
pD3
MW = 106 kDa
Rg = 44.79 Å
S6
15.7 ml
14.8 ml
Vr=11.694
MW=132.9
Rg=39.276
D4
D4_1
MW = 45 kDa
Rg = 30.68 Å
S200
15.1 ml
15.4 ml
Vr=8.187
MW=92.9
Rg=39.743
nsEM
D4_2
MW = 136 kDa
Rg = 47.91 Å
S6
15.4 ml
14.5 ml
Vr=11.243
MW=125.8
Rg=42.789
D4_3
MW = 134 kDa
Rg = 46.53 Å
S6
15.4 ml
14.9 ml
Vr=9.973
MW=161.5
Rg=54.893
D4_4
MW = 199 kDa
Rg = 48.33 Å
S6
14.9 ml
14.7 ml
Vr=14.352
MW=144
Rg=50.272
D5
D5_1
MW = 409 kDa
Rg = 55.03 Å
S6
13.9 ml
13.6 ml
Vr=10.556
MW=408.3
Rg=59.134
nsEM
D5_2
MW = 411 kDa
Rg = 56.92 Å
S6
13.9 ml
13.6 ml
Vr=12.863
MW=426.5
Rg=63.573
pseudoD6
pD6
MW = 121 kDa
Rg = 52.72 Å
S6
15.5 ml
15.1 ml
Vr=33.041
MW=121.3
Rg=38.357
cryoEM
D8
D8_1
MW = 255 kDa
Rg = 64.46 Å
S6
14.5 ml
14.6 ml
Vr=19.824
MW=214.5
Rg=77.931
nsEM
D8_2
MW = 247 kDa
Rg = 62.93 Å
S6
14.6 ml
15.4 ml
nsEM
D8_3
MW = 472 kDa
Rg = 61.41 Å
S6
13.7 ml
13.8 ml
Vr=19.668
MW=403.0
Rg=77.730
Absorbance
Elution volume
log(I(q))
q (A-1)

Figure S3: Detail of the library of axle parts for the design of protein machines with corresponding symmetry, design nomenclature, oligomeric mass (MW) and radius of gyration (Rg) of model, SEC chromatograms, SAXS traces, PDB model, and 3D electron density reconstruction from electron microscopy analysis. For each SEC trace, the predicted elution volume corresponding to the correct oligomer state is given in milliliters in black next to the chromatogram, and the experimental elution volume obtained by fitting the peak is reported in blue (All reported in Table S1). Experimental SAXS traces are shown in the red line, while the theoretical trace corresponding to the design is shown in grey. Volatility ratios (Vr), predicted molecular weight (MW) and radius of gyration (Rg) were computed and shown with each trace, and reported in Table S2. nsEM: data obtained using negative stain electron microscopy processed in symmetry mode; cryoEM: data obtained using single particle cryoelectron microscopy processed in symmetry mode.

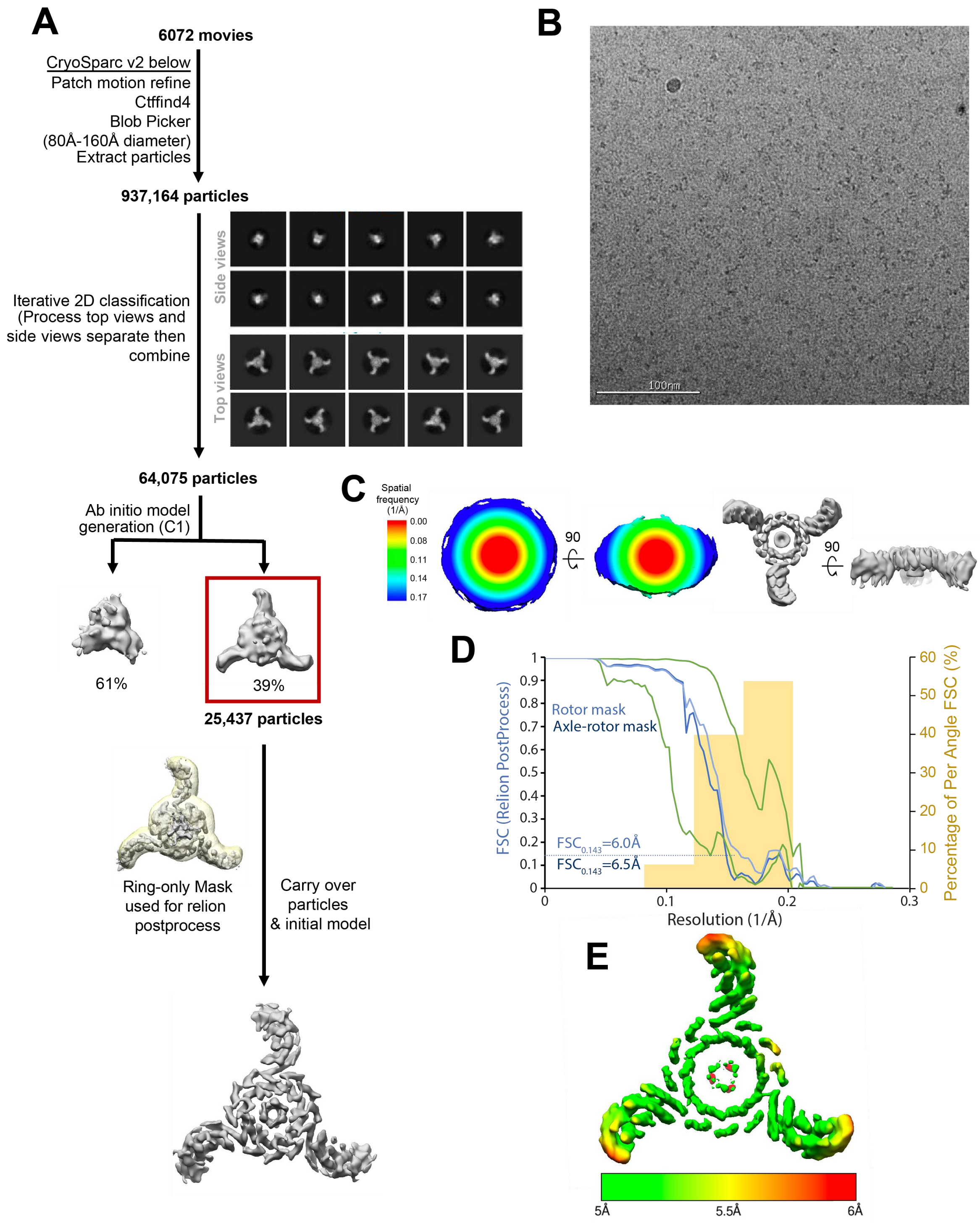

Figure S4: CryoEM data processing pipelines used to generate electron density and structures of the C3-C3 rotary machine. (**A**) Detail of the data processing pipeline (**B**) Representative cryoEM micrograph (**C**) 3D FSC volume calculated for the full axle-rotor assembly (bottom) and colored by radius, which corresponds to information at higher spatial frequency. Sphericity is 0.912 out of 1, indicating anisotropy in one direction. (**D**) Global FSC calculation of fully-masked axle-rotor assembly, and mask for the rotor only with Relion postprocess. Overlaid histogram from output in panel C, showing a distribution of resolutions achieved by a random sampling of views. Green traces correspond to

extremes of the data shown in the histogram. (**E**) Electron density map with corresponding estimated local resolution.

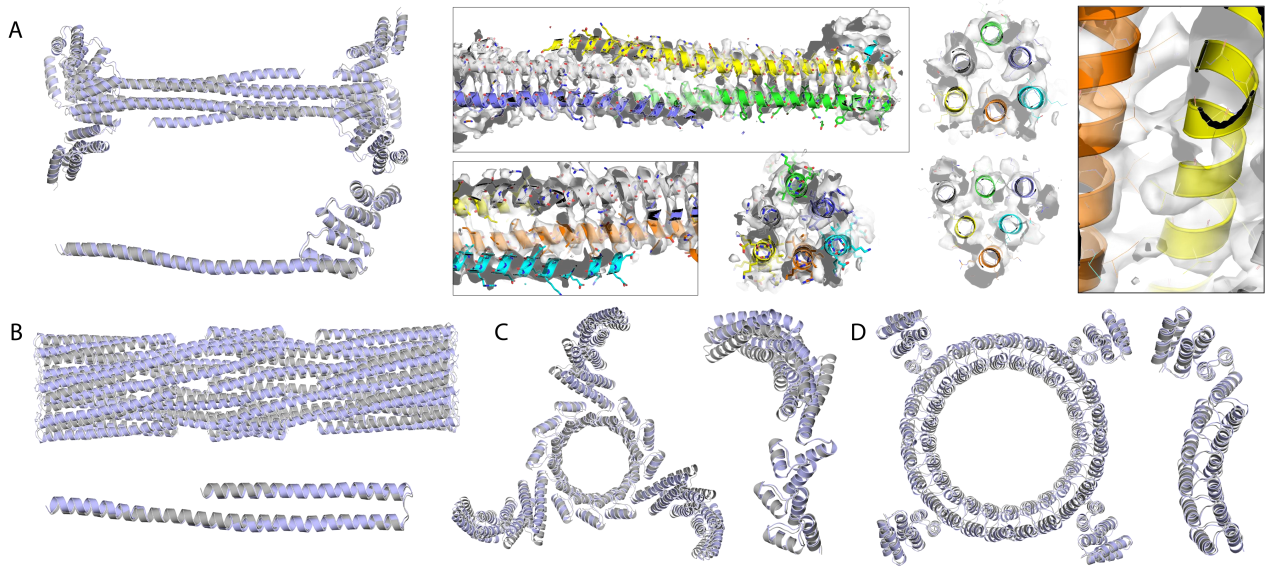

Figure S5: Detailed comparison of designs versus high resolution cryoEM structures. The designs were relaxed into experimental cryoEM electron densities using Rosetta FastRelax and SetupForDensityScoring. (**A**) D3 axle design (D3_3); (Left) Superposition of the designed backbone (grey) and backbone relaxed into the experimental electron density (light blue), full structure and single chain alignment. The computed backbone atom RMSD between the designed and experimental structure is 1.930Å. (Right) Detail of side chain density that becomes visible at this resolution (4.2Å). (**B**) D8 axle design (D8_1). Superposition of the designed backbone (grey) and backbone relaxed into the experimental electron density (light blue), full structure and single chain alignment. The computed backbone atom RMSD between the designed and experimental structure is 2.879Å. (**C**) C3 rotor design (C3_R1). Superposition of the designed backbone (grey) and backbone relaxed into the experimental electron density (light blue), full structure and single chain alignment. The computed backbone atom RMSD between the designed and experimental struringcture is 3.451Å. (**D**) C4_1 rotor design (C4_1). Superposition of the designed backbone (grey) and backbone relaxed into the experimental electron density (light blue), full structure and single chain alignment. The computed backbone atom RMSD between the designed and experimental structure is 1.775Å.

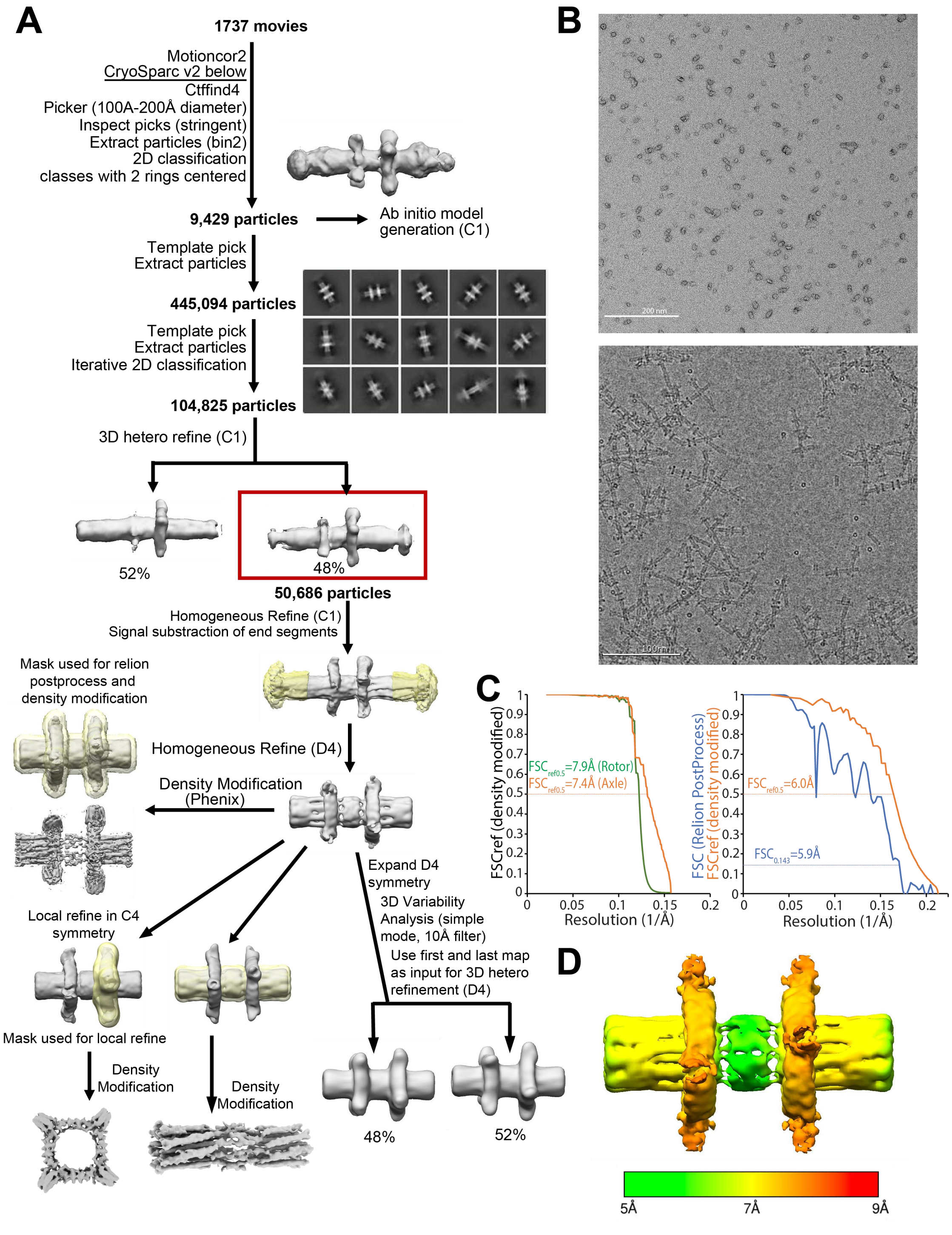

Figure S6: CryoEM data processing pipelines used to generate electron density and structures of the D8-C4 rotary machine. Interestingly, this design self-assembled into higher-order fiber-like structures upon freezing, which highlights the unintended effects of cryogenic conditions on protein assemblies. We could however verify that this fiber assembly did not happen at room temperature in solution, as can be seen from SEC in fig. S10, as well as negative stain EM. (**A**) Detail of the data processing pipeline (**B**) Representative cryoEM micrograph. Top: Negative stain; bottom: cryoEM. Freezing conditions seemed to induce fiber formation via end-to-end contact of the D8 axle. (**C**) FSC

validation curve of axle and rotor alone (left) and axle-rotor full assembly (right) (**D**) Electron density map with corresponding estimated local resolution.

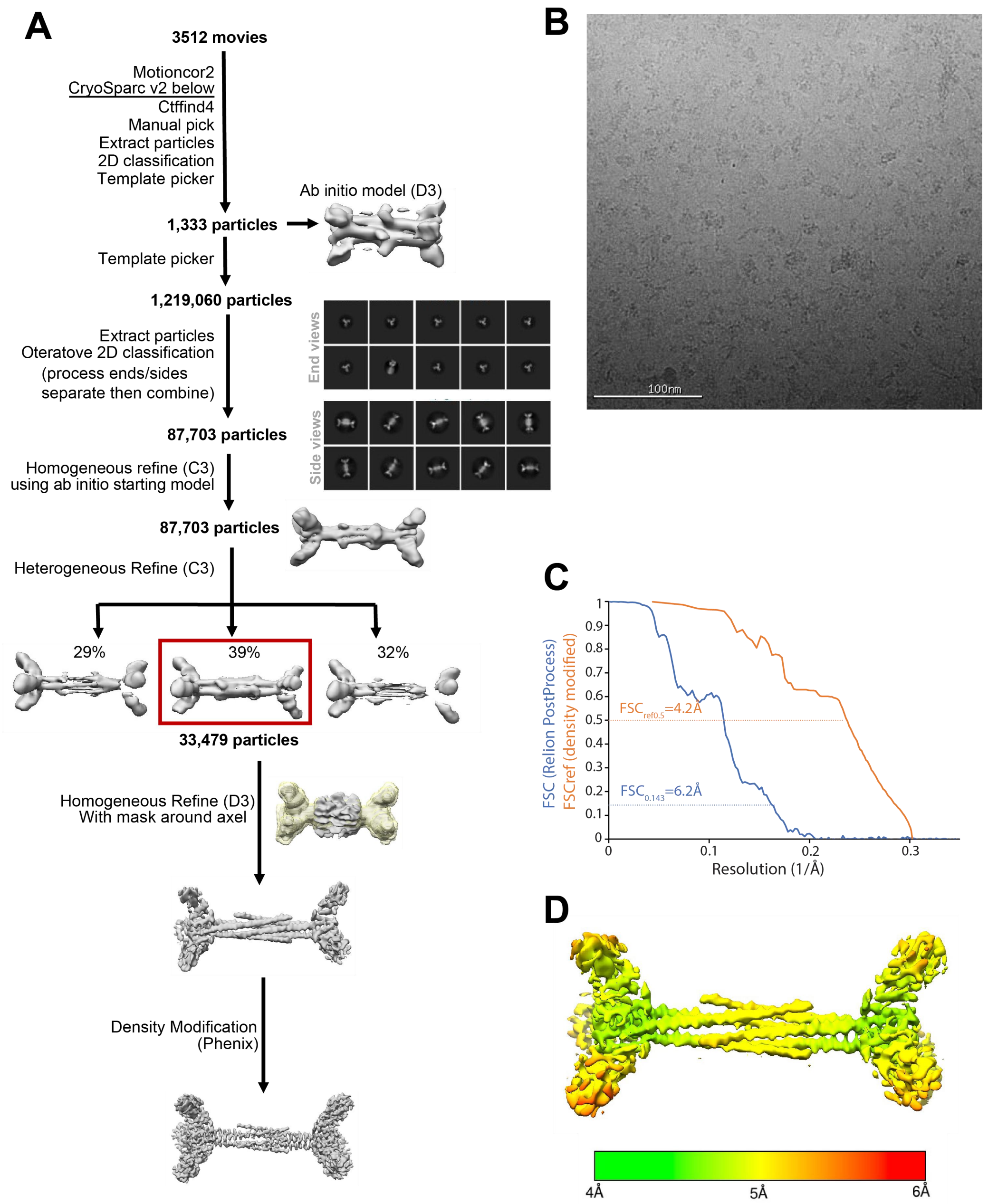

Figure S7: CryoEM data processing pipelines used to generate electron density and a structure of the D3 axle. This data was collected on a version of the D3-C5 rotary machine, for which the C5 rotor did not have arms extension, thus precluding obtention of clear rotor density. We used this dataset to thus focus on obtaining a clear picture of the axle, as detailed here. (**A**) Detail of the data processing pipeline (**B**) Representative cryoEM micrograph (**C**) FSC validation curve (**D**) Electron density map with corresponding estimated local resolution.

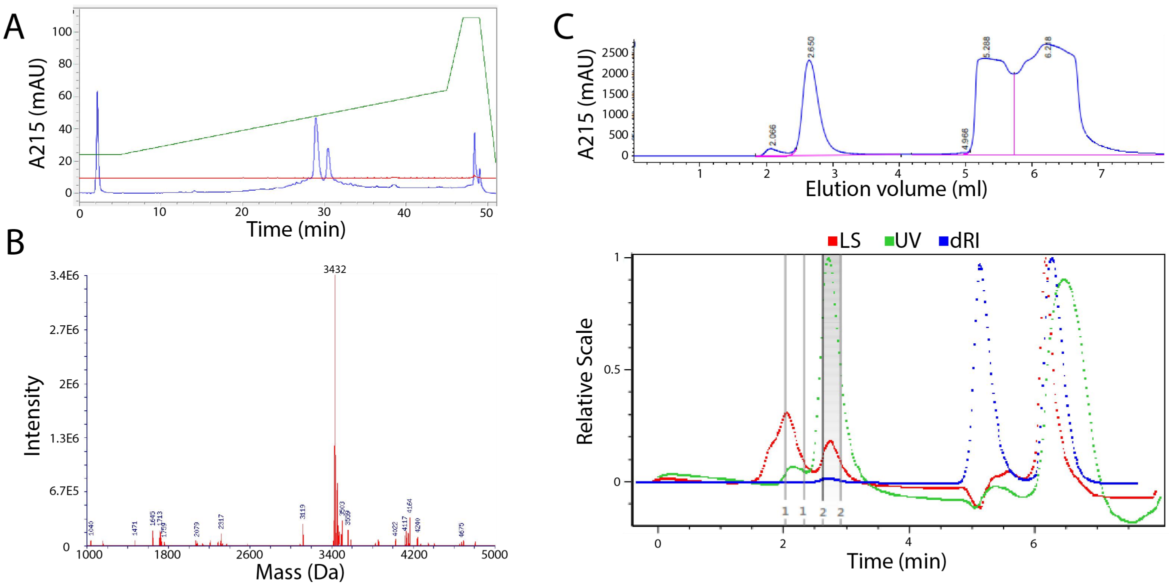

Figure S8: Chemical synthesis of a 36 residue helical peptide self-assembling into a D3 homohexamer (D3_1). (**A**) HPLC chromatogram post synthesis, showing two elution peaks. (**B**) Deconvoluted native mass spectra corresponding to the HPLC peaks. (**C**) Size exclusion chromatography (top, 215nm absorbance) coupled with multiple angle laser light scattering analysis (bottom) of the collected fractions post synthesis and purification. Integration of peak two gives a molecular weight of 25 kDa +/- 7, corresponding to the size of the homohexamer assembly.

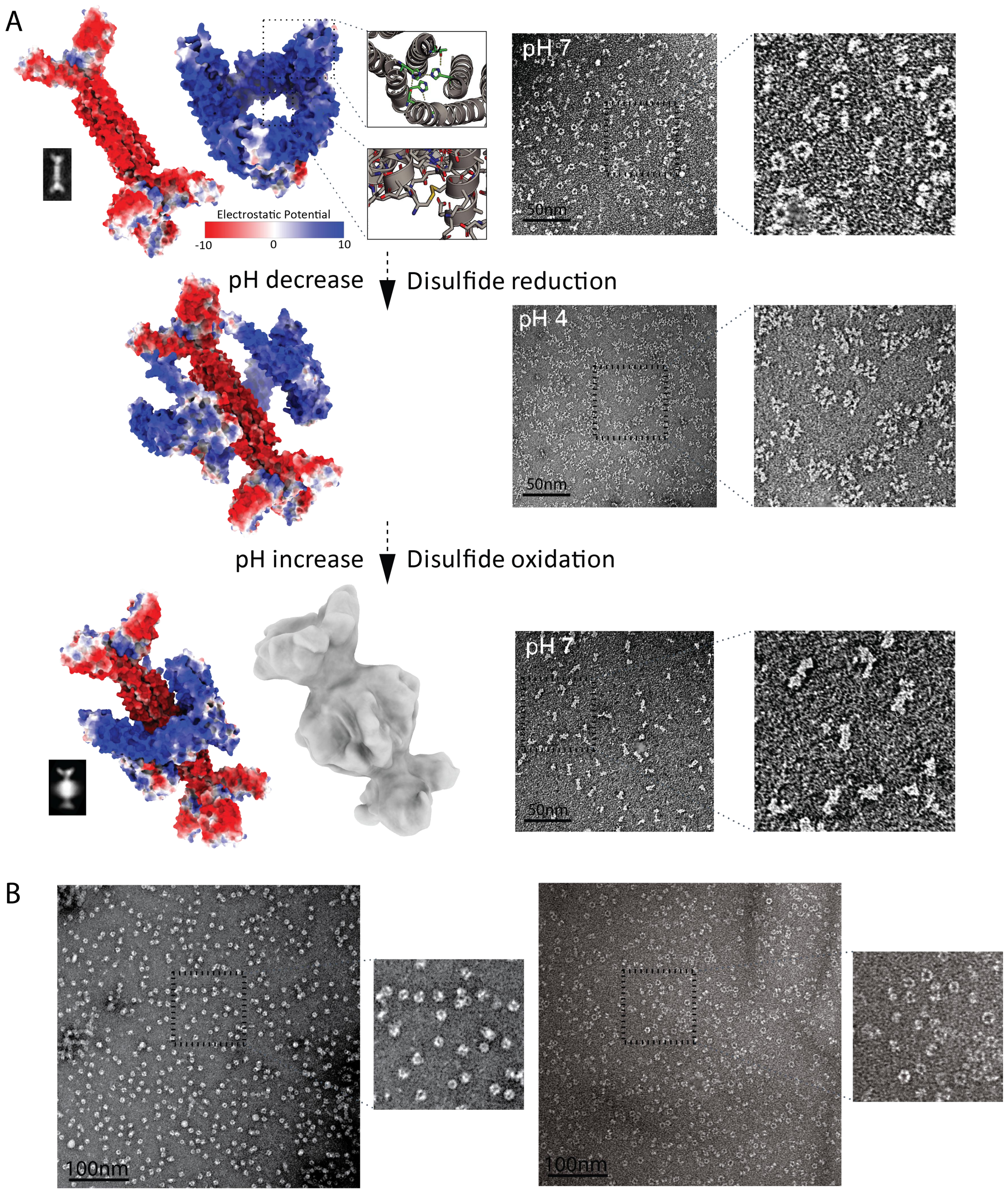

Figure S9: Quasisymmetric axle-rotor complex directed self-assembly strategy. (A) D3-C3 and D3-C5 axles and rotors are designed with complementary charged residues at their interfaces (electrostatic potential rendered from red to blue), buried histidine bond networks and disulfide bonds across the rotor asymmetric unit interfaces to allow pH controlled assembly and oxidoreductive locking of the rotor around the axle. The assembly is here monitored by negative stain EM (square panels on the right with zoomed in windows) while the pH and oxidoreductive state of the medium is modified to yield fully assembled rotors after the assembly process (cryoEM electron density on bottom-middle, and example corresponding 2D class average of the axle alone or axle-rotor assembly). (**B**) The rotor components alone can follow the same pH and oxidoreductive procedure and fully reassemble into homooligomeric assemblies in the absence of the axle. Left: C3 rotor, right: C5 rotor.

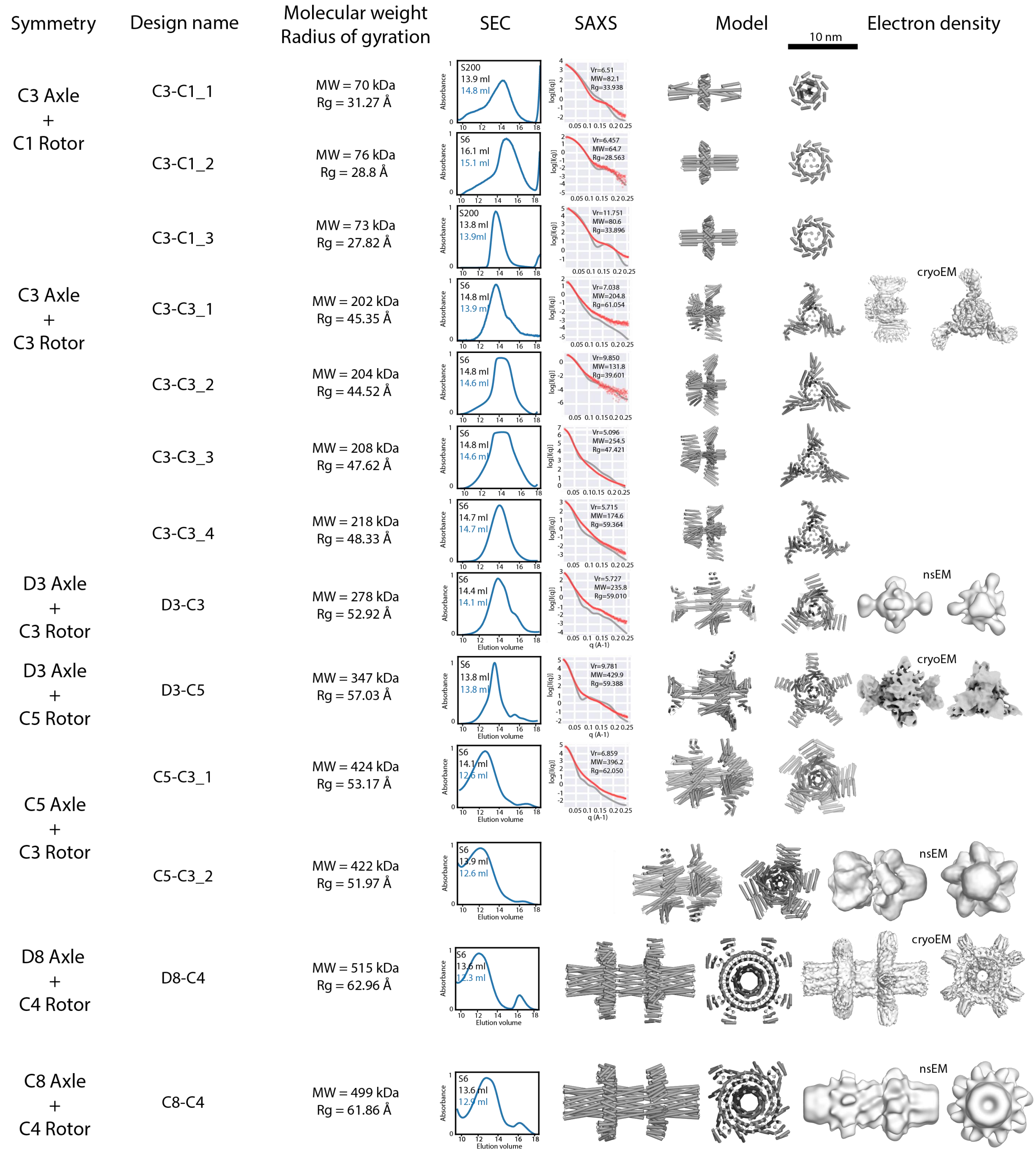

Figure S10: Detail of the library of axle-rotor assemblies with corresponding symmetry, design nomenclature, oligomeric mass (MW) and radius of gyration (Rg) of model, SEC chromatograms, SAXS traces, PDB model, and 3D electron density reconstruction from electron microscopy analysis. For each SEC trace, the theoretical elution volume corresponding to the correct oligomer state is given in milliliters in black next to the chromatogram, and the experimental elution volume obtained by fitting the peak is reported in blue. Experimental SAXS traces are shown in the red line, while the theoretical trace corresponding to the design is shown in grey. Volatility ratios (Vr), predicted molecular weight (MW) and radius of gyration (Rg) were computed and shown with each trace, and reported in

Table S1. nsEM: data obtained using negative stain electron microscopy processed in symmetry mode; cryoEM: data obtained using single particle cryoelectron microscopy processed in symmetry mode.

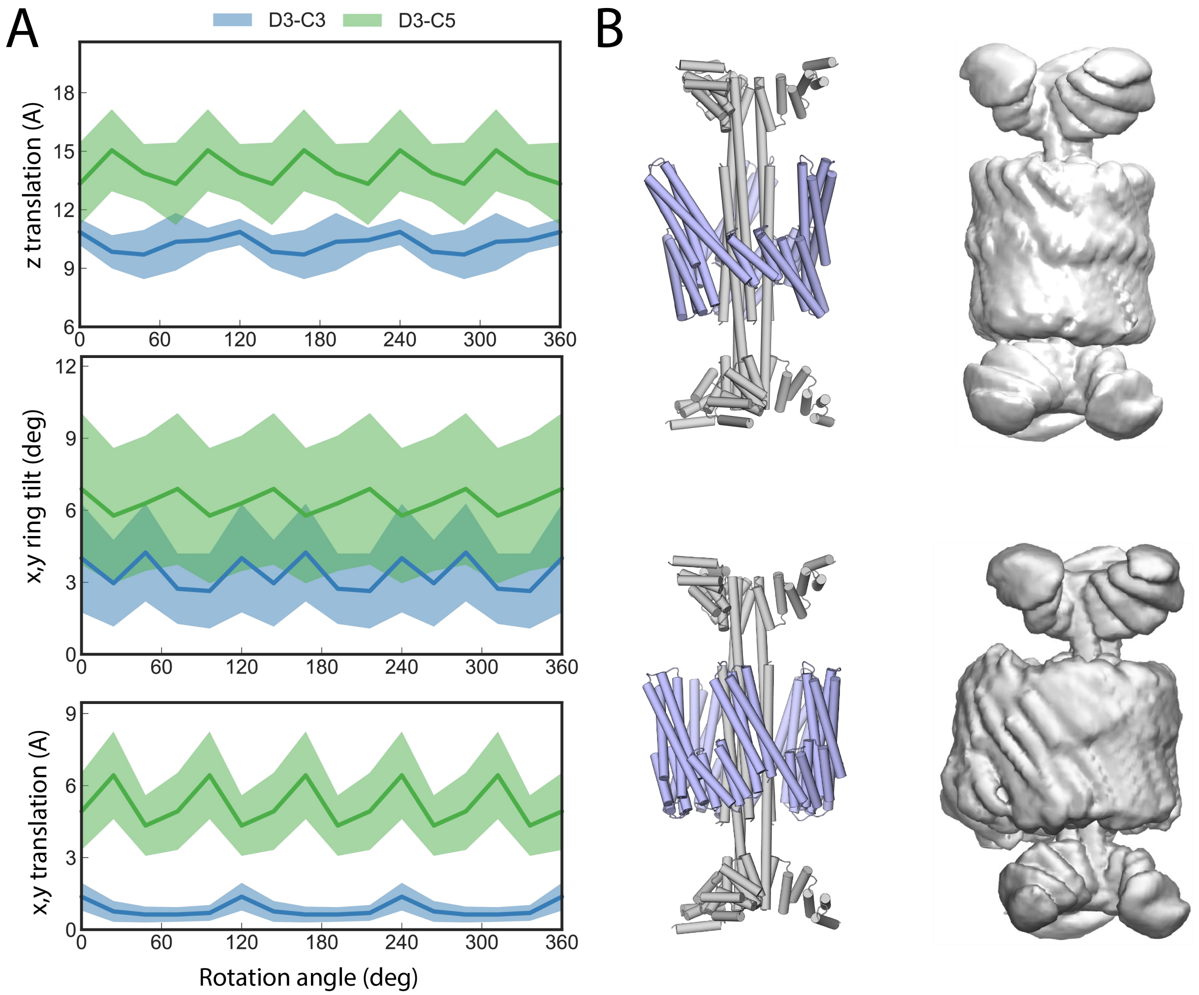

Figure S11: Molecular dynamics simulations performed on D3-C3 and D3-C5 axle-rotor assemblies to investigate the DOF of motion (**A**) The interface shape, size and symmetry of these two design results in different DOFs: the D3-C3 was found to rotate along the z axis, while the D3-C5 rotor showed rotation along x, y and z, as well as translation in z and y. The top panel shows z translation of rotors relative to rotation, the middle panel shows the x and y rotation or the rotor, or tilt, relative to rotation and the bottom panel shows the x, y translation of the rotor relative to the rotation around the axle. (**B**) Top: D3-C3; Bottom: D3-C5; Left: PDB models; Right: density maps of the backbone atoms showing averaged motion of rotor and axle relative to each other.

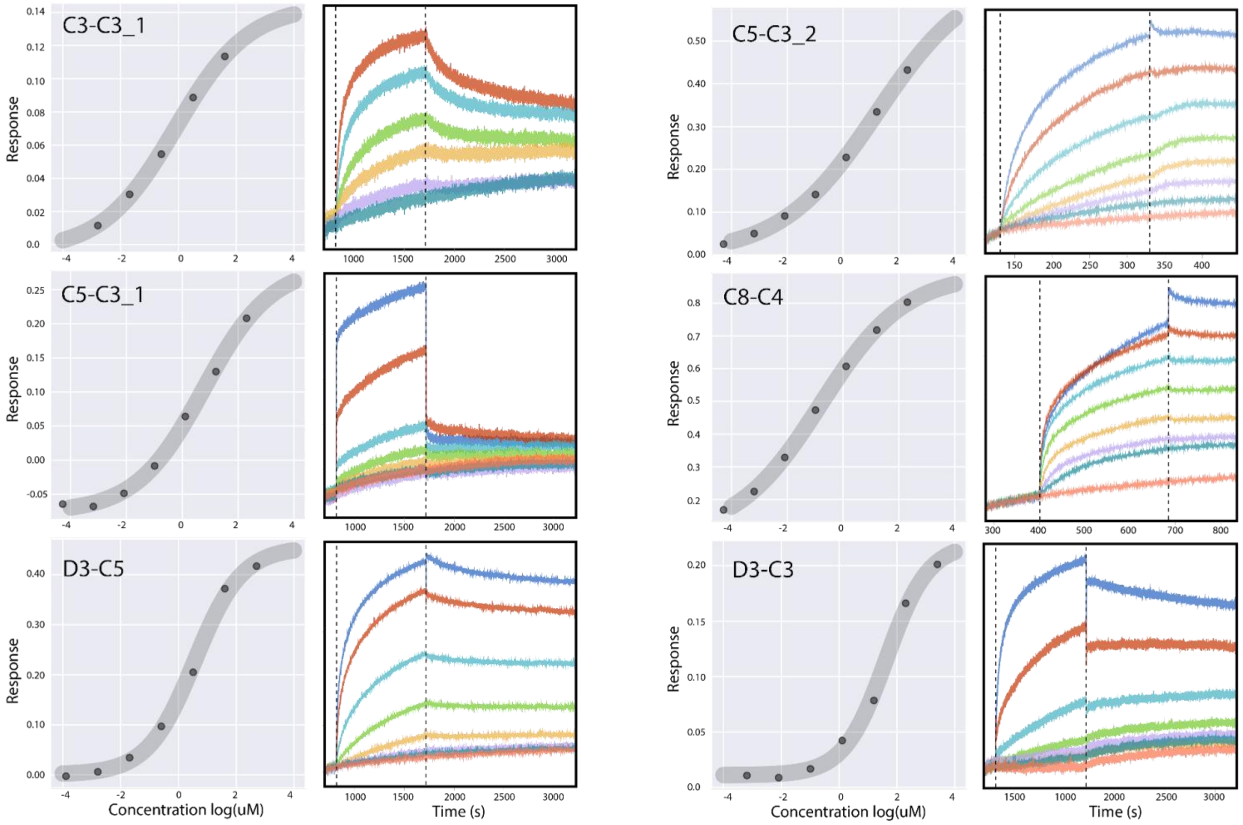

Figure S12: Biolayer interferometry assays measuring *in vitro* binding of rotor and axle parts. For each 6 designs, the equilibrium binding curves from biolayer interferometry binding assays are shown on the left and the corresponding kinetic binding traces shown on the right. Biotinylated axles were immobilized on the tip and the binding in a solution of free rotors was measured. For both D3-C5 and D3-C3, a fresh rotor solution was prepared by buffer exchange from citrate buffer to TBS with reducing agent, and immediately used for binding assays.

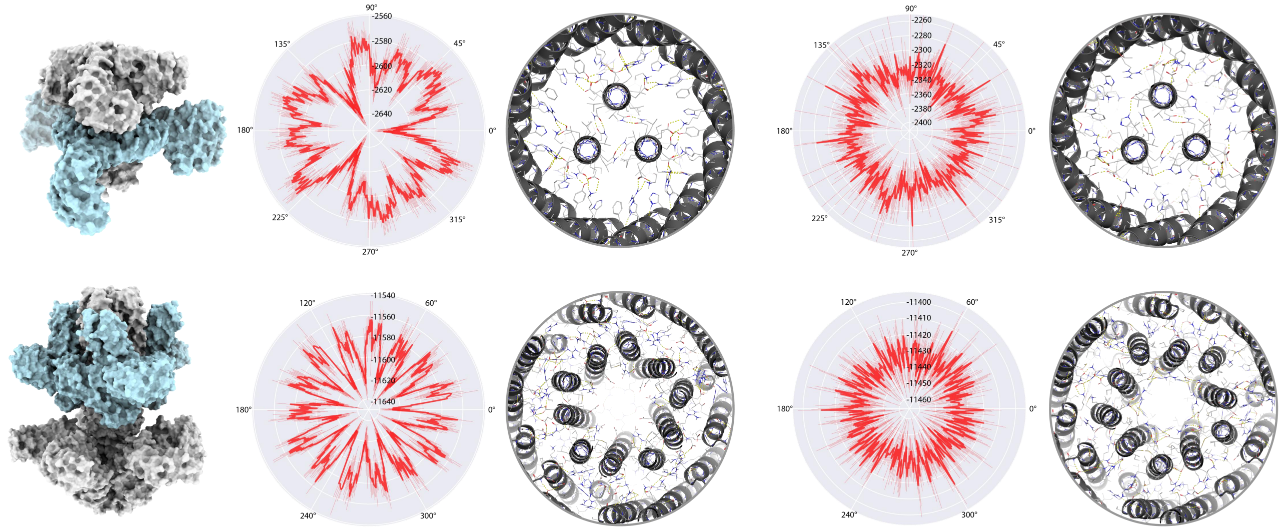

Figure S13: Example of designed energy landscapes. The shape, periodicity and energetics differ drastically depending on the residue identities and contact types for the same protein scaffold. (Top) Two C3-C3 rotors design trajectories corresponding to designs C3-C3_1 and C3-C1_3; (Bottom) Two C5-C3 rotors design trajectories: C5-C3_1, C5-C3_2; (Left) PDB models (rotor color in cyan and axle in grey); (Right) Energy landscapes shown as polar maps, depicting Rosetta Energy Units (REU) vs rotation angle generated by sampling along the rotational degree of freedom while using Rosetta relax, minimization of side chains and scoring for each rotation bins. The mean energy landscape obtained from 10 independent trajectories is shown in red with error bars depicting the standard deviation. The designed interface between axle and rotor at angle=0 is shown beside as cross-sections, showing residue identities and contacts and hydrogen-bond networks (yellow dashed lines) with the helical backbone (shown in grey).

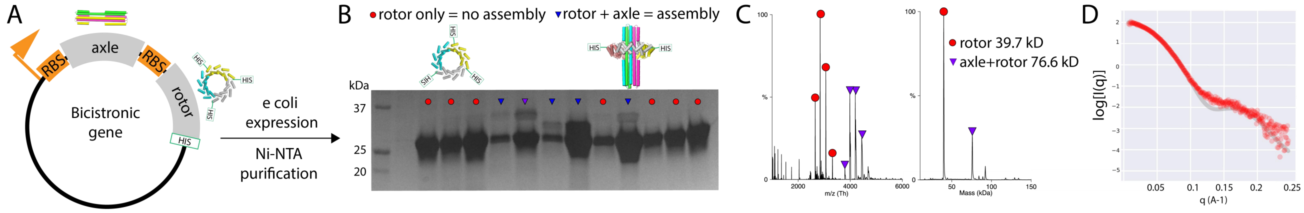

Figure S14: *In vivo* assembly of two component axle-rotor protein assemblies from bicistronically expressed axle and rotor parts (**A**) Plasmid architecture for the bicistronic expression system based pET29b+. (**B**) SDS-PAGE after Ni-NTA purification while bicistronically expressing axle and rotor in the same cell. A single band indicates that the rotor did not pull down the axle (lanes marked with red circle), while 2 bands indicate assembly of axle and rotor (marked as an triangle, purple triangle marks the selected design) (**C**) Convoluted and deconvoluted native mass spectra of the isolated C3-C3 axle-rotor assembly. (**D**) SAXS traces of the purified protein. The experimental trace is shown in the red line, while the theoretical trace corresponding to the design is shown in grey.

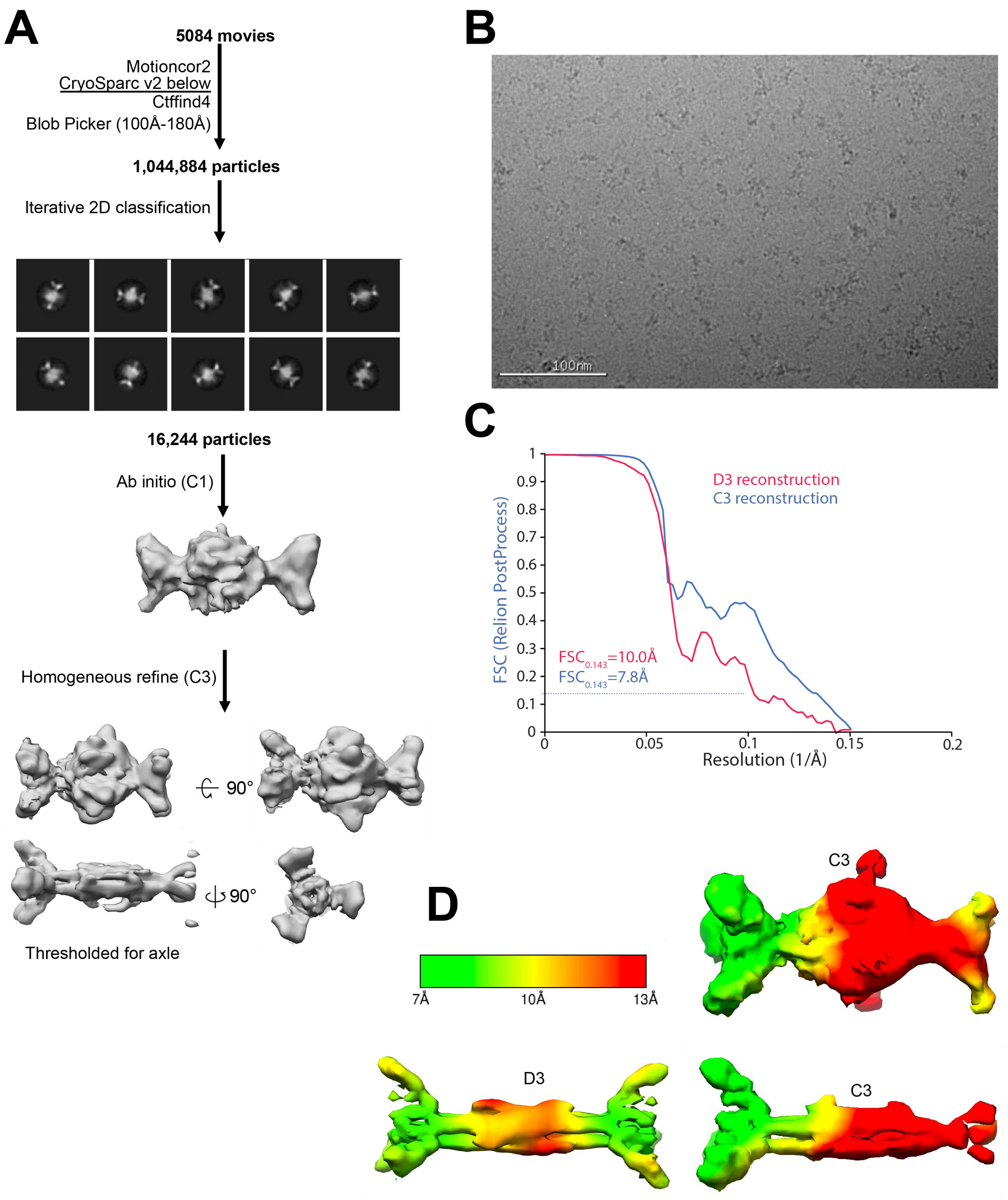

Figure S15: CryoEM data processing pipelines used to generate electron density and structures of the D3-C3 axle-rotor assembly. (**A**) Detail of the data processing pipeline. The C1 reconstruction yielded C3 features, which allowed us to further process the design with C3 symmetry imposed here (the C1 map can be found in Data S1). The C3 reconstruction also showed rotor density that was polar, consistent with the rotor design. The rotor density looks very similar when processed in D3, while yielding a better model for the axle (which has D3 symmetry by design). Therefore we used a whole model processed in D3 mode to present in figure 3C, which is closest to the actual symmetry and structure of the model (**B**) Representative cryoEM micrograph. (**C**) FSC validation curve for C3 and D3

reconstruction of the full axle-rotor assembly. (**D**) Electron density map with corresponding estimated local resolution: axle and rotor in C3, axle alone in D3 and C3 symmetry.

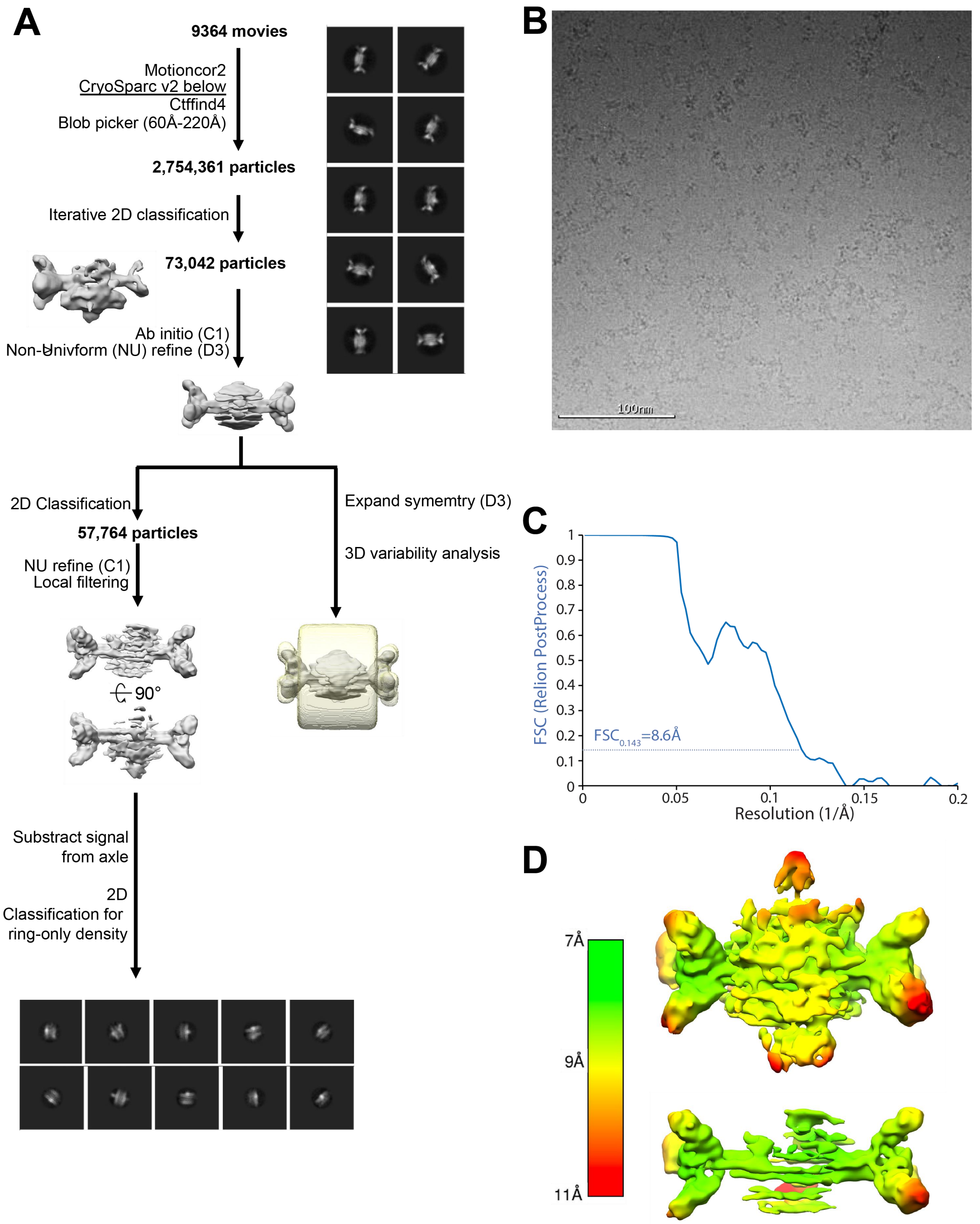

Figure S16: CryoEM data processing pipelines used to generate electron density and structures of the D3-C5 axle-rotor assembly. (**A**) Detail of the data processing pipeline (**B**) Representative cryoEM micrograph (**C**) FSC validation curve of C1 reconstruction (**D**) Electron density map with corresponding estimated local resolution.

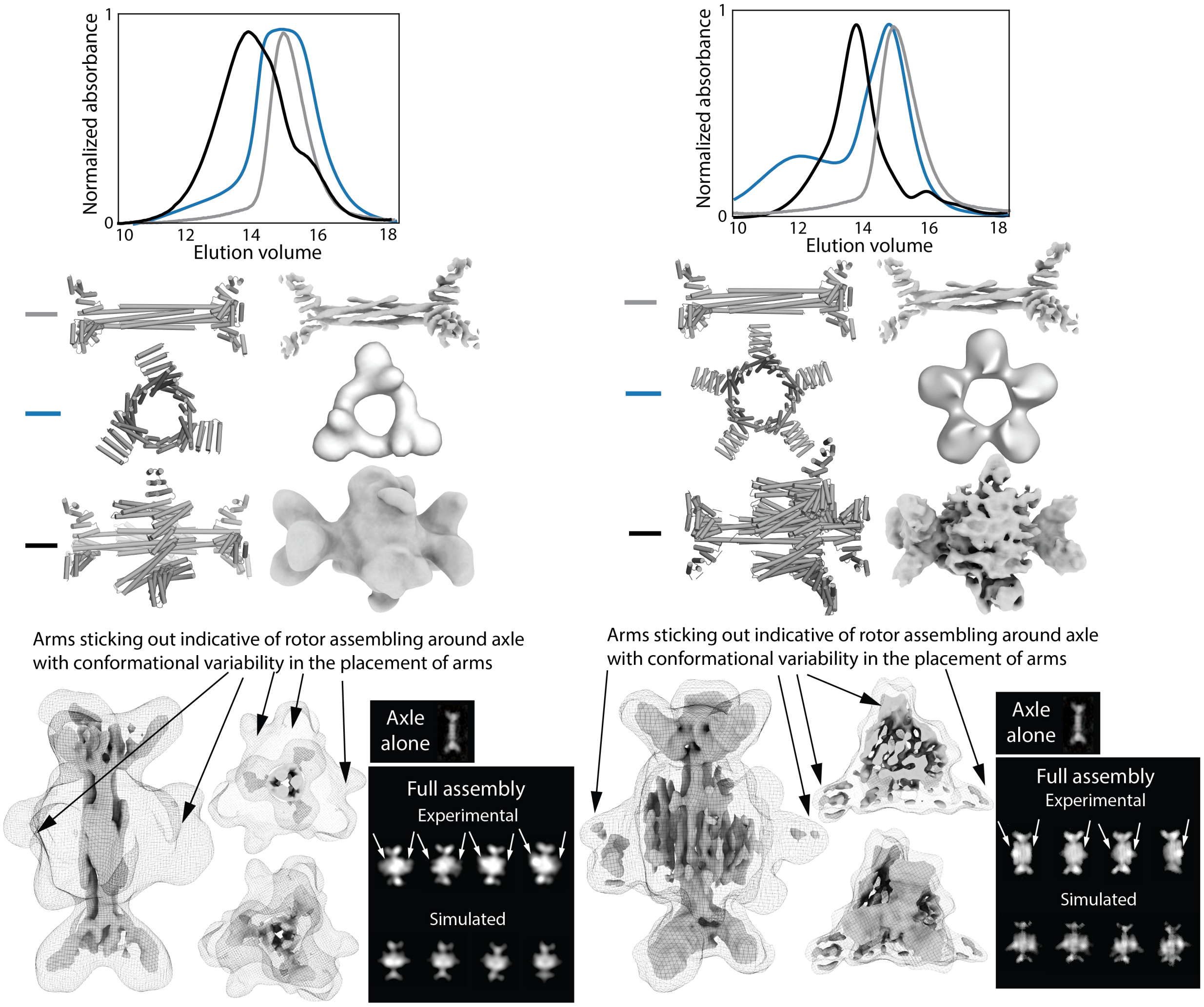

Figure S17: Detailed comparison of SEC traces of fully assembled axle-rotor protein assemblies in the context of electron microscopy data. From left to right: D3-C3 and D3-C5 axle-rotor assemblies. (Top) Overlaid SEC traces corresponding to axle (grey), rotor (turquoise), and axle-rotor assembly (black). (Bottom) PDB model and experimental EM electron density for axle, rotor, and axle-rotor assembly.The SEC analysis shows a clear shift obtained after assembly of axle and rotor compared to axle and rotor alone in the context of the cryoEM data, is evidence for the correct oligomeric assembly state. The D3 axle has a mass larger than the C3 rotor and clear "arms"-like features, this is clearly visible in the simulated projections, which clearly match the experimental data. 2D averages and 3D reconstruction clearly capture secondary structure (helices and "arms") but could not be fully resolved for the rotor, consistent with conformational variability of the rotor relative to the axle. For these two designs D3-C3 and D3-C5, conformational variability is most pronounced due to the internal degrees of freedom of the assembly. Unlike the C3-C3 design in which the rotor component dominates the alignment signal, for these two designs the D3 axle signal dominates the alignment (this is due to the axle presenting always the same side view and having greater mass, aspect ratio and more features than the rotor). In the case of the D3-C3 axle-rotor assembly, the axle can be fully resolved while the rotor is partially resolved, which is indicative of conformational variability, consistent with an even flatter energy landscape and more

degrees of freedom than previous assemblies. The 3D CryoEM reconstruction of the D3 axle alone, or assembled with C3 in the D3-C3 axle-rotor assembly (left) at 7.8Å resolution suggests that the rotor sits midway across the D3 axle, and shows the main features of the designed structure and density for rotor and axle, evident in the experimental 2D class average panel (middle) compared to simulated 2D class average (bottom). In the case of the D3-C5, once again the SEC analysis shown above shows a clear shift obtained after assembly of axle and rotor compared to axle and rotor alone in the context of the cryoEM data, is evidence for the correct oligomeric assembly state. The 3D CryoEM reconstruction of the D3 axle alone or assembled with the C5 rotor is shown as experimental data (top row) and simulated 2D class averages (bottom row). The D3-C5 rotor cryoEM electron density at 8.6Å captures the features of the designed structure also evident in the class averages. For both axle-rotor designs, the 2D averages and 3D reconstruction clearly capture secondary structure (helices and rotor “arms” around the axle) corresponding to the rotor but could not be fully resolved, consistent with conformational variability of the rotor relative to the axle. In the manuscript, we interpret the fact that one component can be resolved while the other can't as indicative of conformational variability. In a case with a clear axle structure but blurry rotor density, the alternative possibility is that the disordered component is somehow damaged / unfolded / misassembled, we consider this unlikely because: the simulated projections for the particles match the experimental 2D class average (as seen above); SAXS, SEC and negative stain EM data indicate that the axle-rotor assemblies have a monodisperse size corresponding to the correct oligomeric state as shown above. We added a comprehensive supplementary figure comparing SEC assembly and EM data, shown here above; The axle-rotor assembly is very different from the axle and rotor alone in negative stain and cryo EM data (figure above showing the axle alone vs in assembly). No single rotor components can be found in the images or classes of axle-rotor samples after the assembly process (figure below). This is consistent with the rotor being trapped about the axle; axle-rotor assemblies can be left at 4C for weeks, and further size exclusion chromatography analysis and EM shows that the assemblies are not disassembling, again indicating that both components are fully assembled and locked together by the disulfide staples; when the axles are not present and the assembly process is performed (pH and oxido-reductive process), the rotors appear fully reassembled by negative stain (fig. S9). The EM data is clearly different when both components are assembled together, and we can see structural features corresponding to the rotor arms, as well as helical secondary structure corresponding to the rotor as shown above and on the left; we were able to obtain density for the rotor after masking the axle signal (see fig. S16).

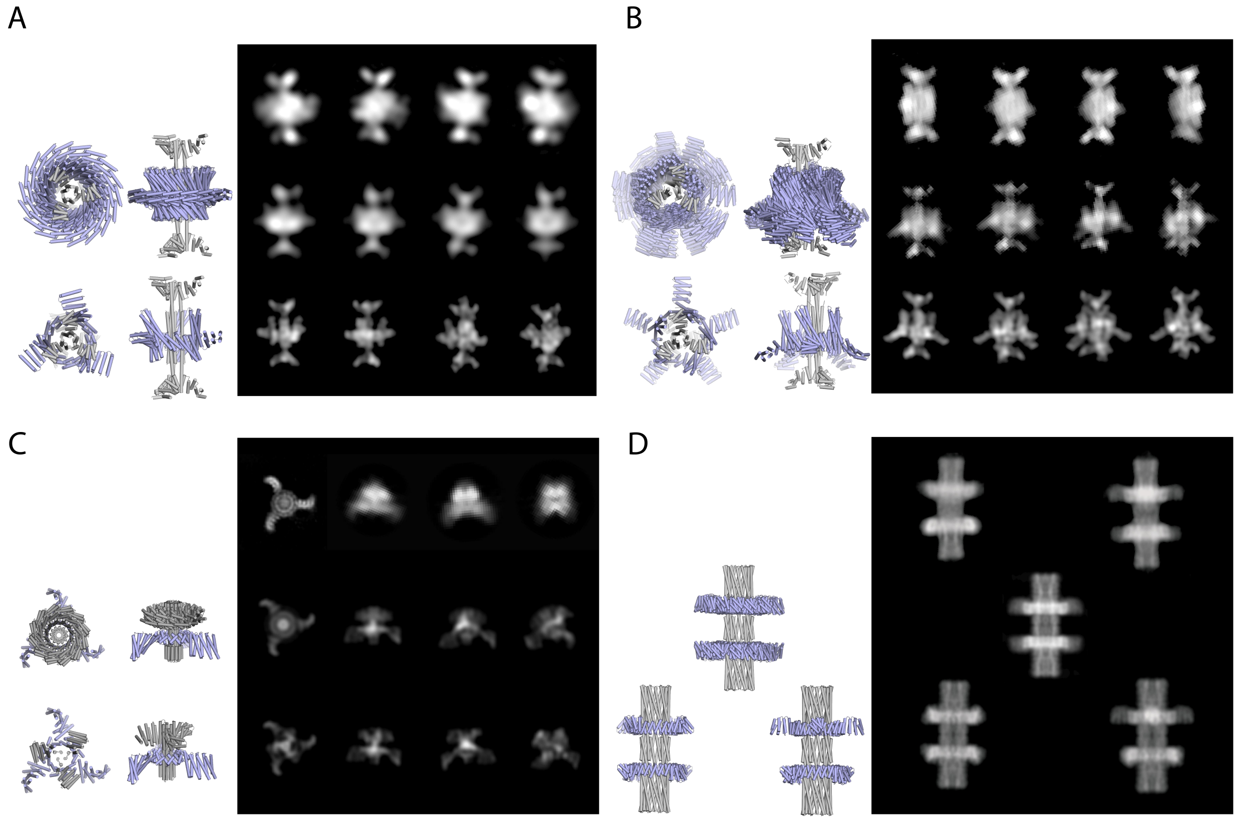

Figure S18: Detail of DOF sampling for the generation of the theoretical cryoEM 2D class averages projections compared to experimental data and models. (**A**) D3-C3 axle-rotor (top) Experimental 2D averages (middle) Projections obtained when taking into account the rotational DOF and simulating 10 trajectories with corresponding PDB model shown on the left (bottom) Projections obtained when not taking into account the rotational DOF and simulating 10 trajectories with corresponding PDB model shown on the left. (**B**) D3-C5 axle-rotor (top) Experimental 2D averages (middle) Projections obtained when taking into account the rotational and translational DOF and simulating 10 trajectories with corresponding PDB model shown on the left (bottom) Projections obtained when not taking into account the rotational and translational DOFs and simulating 10 trajectories with corresponding PDB model sho1wn on the left. (**C**) C3-C3 axle-rotor (top) Experimental 2D averages (middle) Projections obtained when taking into account the rotational DOF and simulating 10 trajectories with corresponding PDB model shown on the left (bottom) Projections obtained when not taking into account the rotational DOF with corresponding PDB model shown on the left. (**D**) D8-C4 axle-rotor (top) Experimental 2D averages (middle) Projections obtained when taking into account the rotational DOF and simulating 10 trajectories with corresponding PDB model shown on the left (bottom) Projections obtained of the two equiprobable rotational states corresponding to the 8 fold energy minima with corresponding PDB models shown on the left. For generating all panels, all projections were low passed to the same resolution.

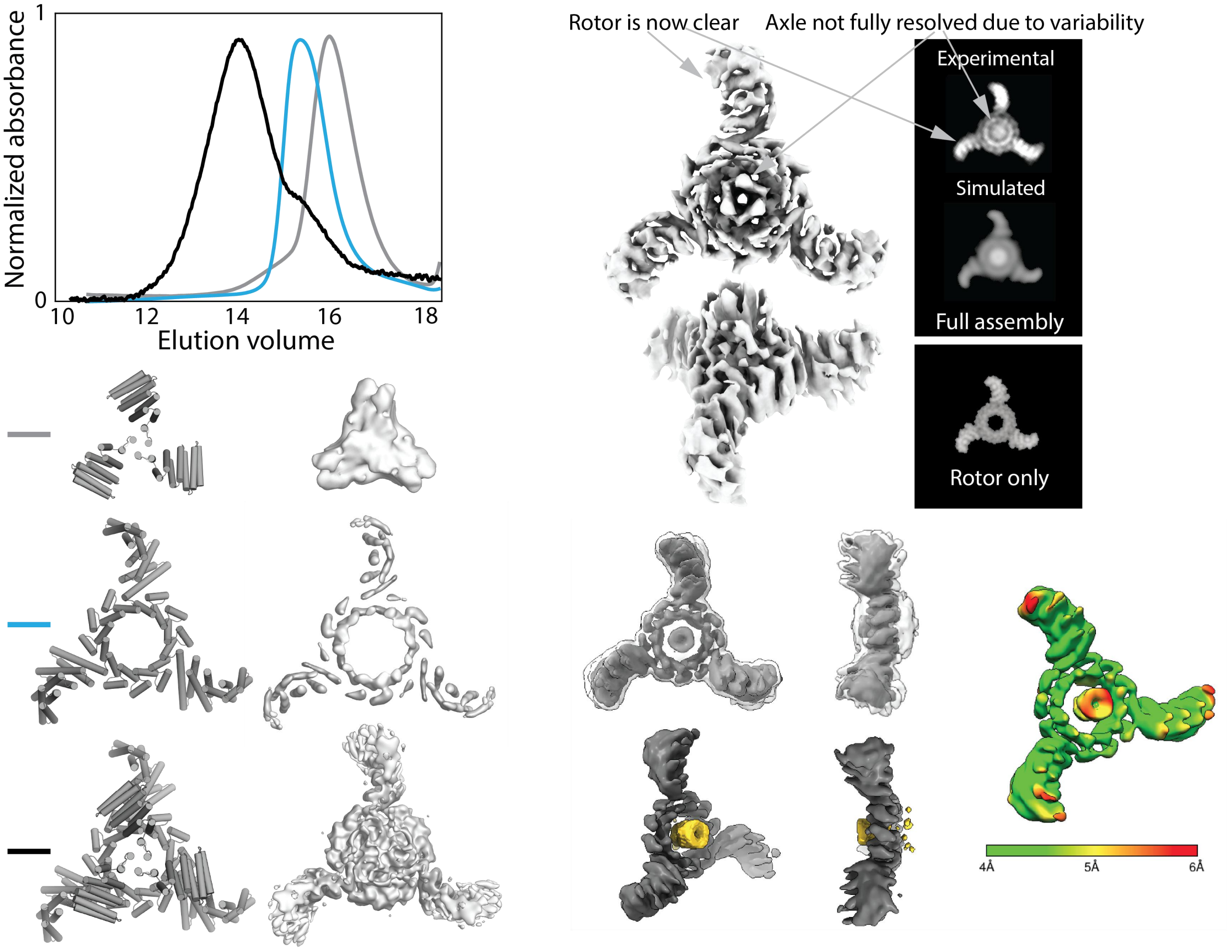

Figure S19: Detailed comparison of SEC traces of C3-C3 axle-rotor protein assembly in the context of electron microscopy data. The SEC analysis shows a clear shift obtained after assembly of axle and rotor compared to axle and rotor alone in the context of the cryoEM data, is evidence for the correct oligomeric assembly state. The cryoEM reconstruction of the C3-C3 axle-rotor assembly (left) at 6.5Å resolution shows the main features of the designed structure and density for rotor and axle, evident in the experimental 2D class average panel (top right) compared to simulated 2D class average (middle right). Simulated projections from a model of the rotor alone, clearly shows that in the absence of the axle the center density is absent. On the bottom right is a global refinement and reconstruction in C1 with a mask for the rotor only, demonstrating that when the rotor is in focus the axel is not. It is clear that the axle is unresolved although density corresponding to it in gold is obvious. The cryoEM data indicates that one of the components, the rotor, can be fully resolved while the axle is partially resolved, which is indicative of conformational variability, consistent with a flatter energy landscape. The C3 rotor has a mass greater than the C3 axle and clear “arms”-like features, which bias the alignment algorithm in favor of the rotor component (*i.e.* particles are aligned on the rotor components and not the axle, generating a blurrier view of the central axle). This is clearly visible in the simulated projections, which clearly match the experimental data. 2D averages and 3D reconstruction clearly capture secondary structure (helices and “arms”) but could not be fully resolved for the axle, consistent with conformational variability of the rotor relative to the axle.

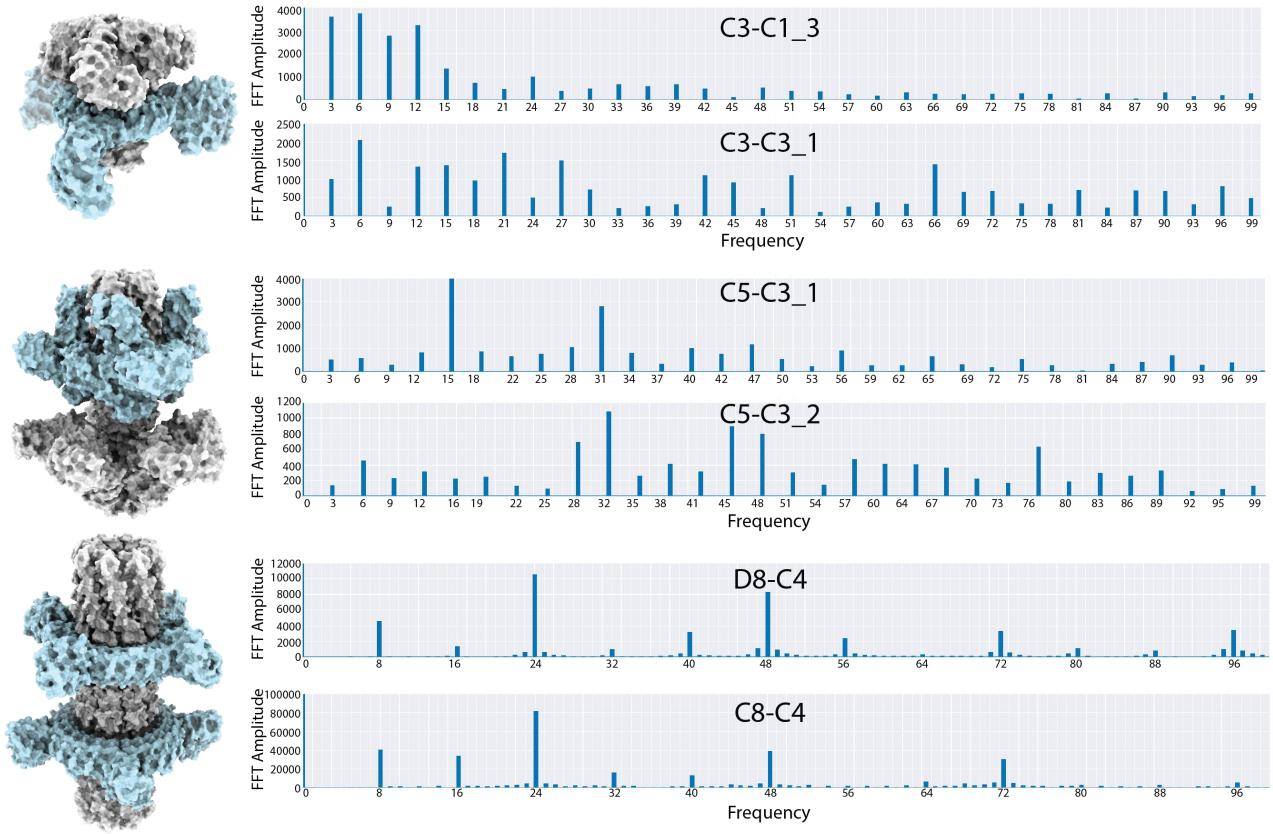

Figure S20: Fourier transform of energy landscapes of shape complementary axle-rotor assemblies. (Top) Two C3-C3 rotors design trajectories corresponding to designs C3-C3_1 and C3-C1_3; (Middle) Two C5-C3 axle-rotor design trajectories: C5-C3_1, C5-C3_2; (Bottom) D8-C4 and C8-C4 axle-rotor assemblies; (Left) PDB models (rotor color in cyan and axle in grey); (Right) FFT signal versus frequency computed from rotational energy landscapes (Rosetta Energy Units (REU) vs rotation angle) generated by sampling along the rotational degree of freedom while using Rosetta relax, minimization of side chains and scoring for each rotation bins.

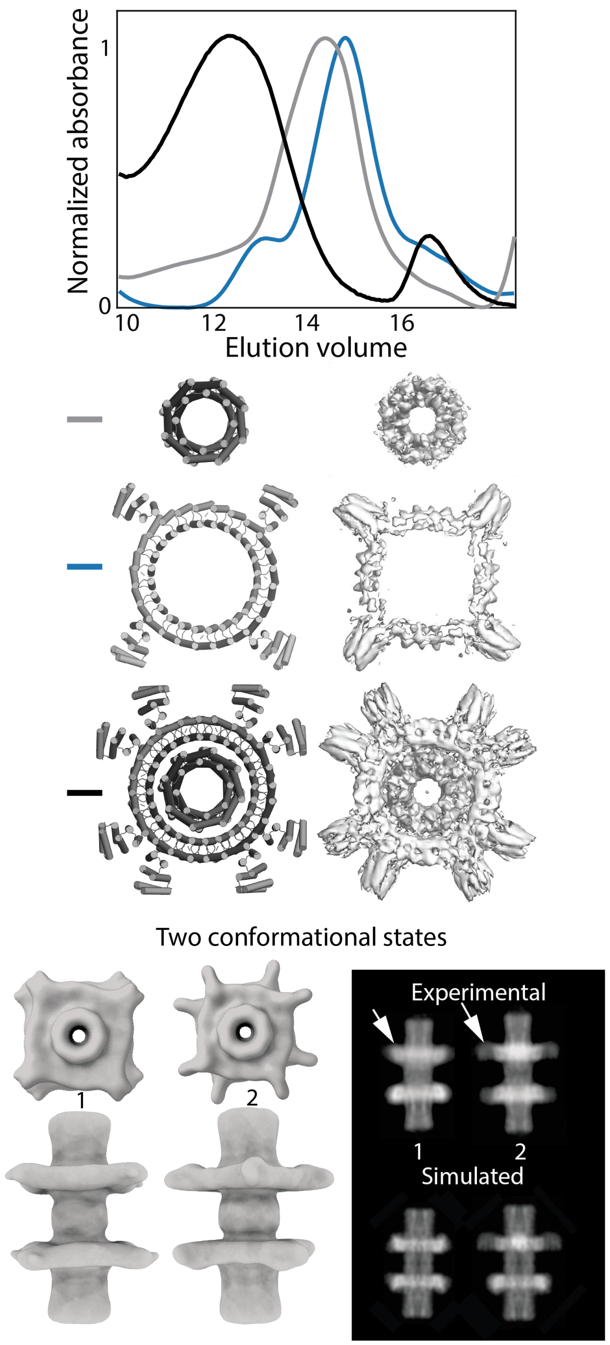

Figure S21: Detailed comparison of SEC traces of D8-C4 protein machines assembly in the context of electron microscopy data. The SEC analysis shows a clear shift in mass measured after assembly of axle and rotor compared to axle and rotor alone, which in the context of the cryoEM data is evidence for the correct oligomeric assembly state. The high symmetry, the clear two states present in the data, and mass of the assembly allowed for efficient alignment of both components. This cryoEM data at 5.9Å resolution shows the main features of the designed structure, evident in the experimental 2D class average panel (top right) compared to simulated 2D class average (bottom right). Both components are clearly resolved in the assembly, which is explained by the assembly sampling only two conformational states: the axle relative to the rotor can be fully resolved in the absence of a degeneracy of conformational states, which is consistent with the designed rugged energy landscape. The variability analysis from this cryoEM

data further supports the existence of two discrete assembly states (bottom left), which is not a direct proof of rotation but rather a collection of frozen snapshots of different assemblies that sample the symmetric energy landscape. By design, our axle-rotor assemblies have multiple symmetrically identical yet physically distinct rotational states, which cannot be distinguished by cryoEM, but they are nonetheless physically distinct, single molecule measurement will be necessary in the future to monitor transitions between them.

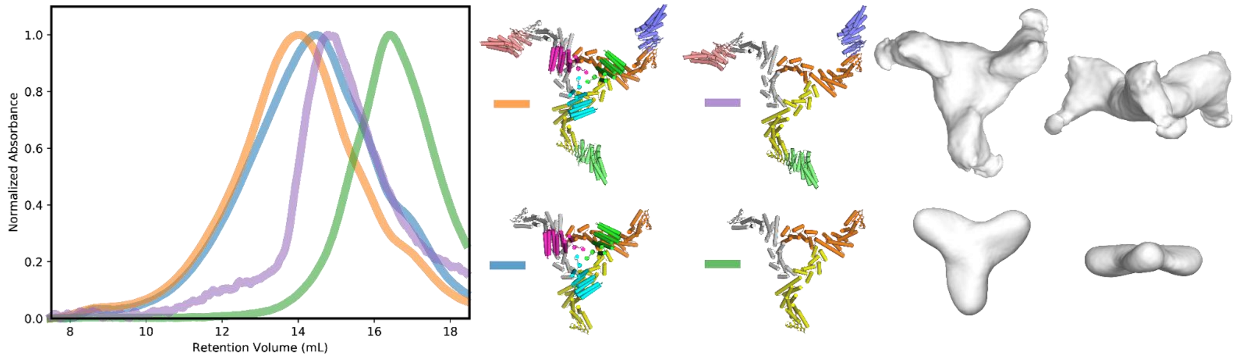

Figure S22: axle-rotor assembly modular extension by systematic fusion with reversible heterodimers. (Left) SEC elution profiles corresponding the C3-C3 rotor assembly with and without heterodimer arms extension, or rotor only with or without heterodimer extension. (Right) Top and side views of negative stain 3D reconstruction corresponding to the rotor only with or without the heterodimer arms.

	SEC Elution volume		
**Model**	**Predicted**	**Experimental**	**% deviation**
**D2_1**	15.9	15.2	4.61
**D2_2**	15.5	15.3	1.31
**D3_1**	16.2	16.1	0.62
**D3_2**	15.2	15.5	1.94
**D3_3**	15.5	15.2	1.97
**D3_4**	15.7	15.6	0.64
**D3_5**	15.7	15.1	3.97
**D3_6**	15.7	15.2	3.29
**D3_7**	15.7	15.6	0.64
**D3_8**	15.7	15.1	3.97
**pD3**	15.7	14.8	6.08
**D4_1**	15.1	15.4	1.95
**D4_2**	15.4	14.5	6.21
**D4_3**	15.4	14.9	3.36
**D4_4**	14.9	14.7	1.36
**D5_1**	13.9	13.6	2.21
**D5_2**	13.9	13.6	2.21
**pD6**	15.5	15.1	2.65
**D8_1**	14.5	14.6	0.68
**D8_2**	14.6	15.4	5.19
**D8_3**	13.7	13.8	0.72
**C3_A1**	15.9	15.9	0
**C3_A2**	15.8	16.3	3.07
**C3_A3**	15.8	15.1	4.64
**C3_A4**	16	16.2	1.23
**C3_R1**	15.6	15.3	1.96
**C3_R2**	15.9	16.3	2.45
**C3_R3**	15.2	14.9	2.01
**C4_1**	15.4	15.1	1.99
**C5_1**	14.5	15	3.33
**C5_2**	14.7	15	2
**C8_1**	14	13.9	0.72
**C3-C1_1**	13.9	14.8	6.08
**C3-C1_2**	16.1	15.1	6.62
**C3-C1_3**	13.8	13.9	0.72

**C3-C3_1**	14.8	13.9	6.47
**C3-C3_2**	14.8	14.6	1.37
**C3-C3_3**	14.8	14.6	1.37
**C3-C3_4**	14.7	14.7	0
**D3-C3**	14.4	14.1	2.13
**D3-C5**	13.8	13.8	0
**C5-C3_1**	14.1	12.6	11.9
**C5-C3_2**	13.9	12.6	10.32
**D8-C4**	13.6	12.3	10.57
**C8-C4**	13.6	12.9	5.43

Table S1: Size exclusion chromatography experimental elution volumes compared predicted from design models. For each design, the predicted and experimental elution volume are reported along with the corresponding deviation.

	Computed from model		Computed from SAXS data		Fit			
Model	MW (kDa)	Rg (A)	MW (kDa)	Rg (A)	Vr	X^2	MW deviation from model (%)	Rg deviation from model (%)
D3_1	21	24.55	20.6	21.233	10.004	0.02	1.9	13.51
D3_2	35	24.55	40.4	27.465	5.245	0.01	15.43	11.87
D3_3	123	62.15	150.9	50.657	6.003	0.09	22.68	18.49
D3_4	105	45.51	122.9	41.681	14.492	1.5	17.05	8.41
D3_5	105	43.97	53.9	41.878	5.715	0.05	48.67	4.76
D3_6	105	45.21	45.9	22.135	8.565	0.13	56.29	51.04
D3_7	106	45.51	68.9	28.852	4.793	0.09	35	36.6
D3_8	107	45.51	92	43.705	3.494	0.08	14.02	3.97
pD3	106	44.79	132.9	39.276	11.694	0.16	25.38	12.31
D4_1	45	30.68	92.9	39.743	8.187	1.66	106.44	29.54
D4_2	136	47.91	125.8	42.789	11.243	0.66	7.5	10.69
D4_3	134	46.53	161.5	54.893	9.973	0.94	20.52	17.97
D4_4	199	48.33	144	50.272	14.352	0.89	27.64	4.02
D5_1	409	55.03	408.3	59.134	10.556	0.1	0.17	7.46
D5_2	411	56.92	426.5	63.573	12.863	0.17	3.77	11.69
pD6	121	52.72	121.3	38.357	33.041	0.02	0.25	27.24
D8_1	255	64.46	214.5	77.931	19.824	5.3	15.88	20.9
D8_3	472	61.41	403	77.73	19.668	1.03	14.62	26.58
C3_A1	89	33.74	97.9	36.38	12.923	0.8	10	7.82
C3_A2	96	34.51	138	39.17	12.067	0.09	43.75	13.5
C3_A3	101	39.41	125.2	43.24	10.217	1.62	23.96	9.72
C3_A4	85	33.02	111.2	38.509	12.225	0.23	30.82	16.62
C3_R1	112	49.37	139.4	41.787	4.684	15.48	24.46	15.36
C3_R2	88	39.41	113.9	37.524	5.184	0.01	29.43	4.79
C3-C1_1	70	31.27	82.1	33.938	6.457	0.1	17.29	8.53
C3-C1_2	76	28.8	64.7	28.563	6.457	0.08	14.87	0.82
C3-C1_3	73	27.82	80.6	33.896	11.751	0.05	10.41	21.84
C3-C3_1	202	45.35	204.8	61.054	7.038	0.81	1.39	34.63
C3-C3_2	204	44.52	131.8	39.601	9.85	0.19	35.39	11.05
C3-C3_3	208	47.62	254.5	47.421	5.096	0.02	22.36	0.42
C3-C3_4	218	48.33	174.6	59.364	5.715	0.12	19.91	22.83
D3-C3	278	52.92	235.8	59.01	9.781	0.2	15.18	11.51
D3-C5	347	57.03	429.9	59.388	9.781	0.14	23.89	4.13
C5-C3_1	422	53.17	396.2	62.05	6.859	0.07	6.11	16.7

Table S2: SAXS metrics and comparison to designed models. Molecular weight (MW) and radius of gyration (Rg) are computed and reported from both the PDB models and the SAXS data, and the fit of the SAXS data to the model is evaluated by computing the Volatility ratio (Vr) and the deviation of molecular weight and radius of gyration from the model. $\chi 2$ values reported in this table were not found to be sufficiently informative to evaluate of goodness of fit.

	C3-C3	D3 axle	D3-C5	D3-C3	D8-C4
**Number of micrographs**	6,072	3,512	9,364	5,084	1,737
**Nominal magnification**	130,000X				36,000X
**Voltage**	300 kV				200 kV
**Electron Fluence**	$90e^-/Å^2$				$65e^-/Å^2$
**Pixel size**	1.05Å				1.16Å
**Defocus range**	-1.7 to -2.8μm	-1.5 to -2.4μm	-1.3 to -2.5μm	-0.5 to -3.8μm	-1.4 to -4.2μm
**EMDB ID**	EMD-25580	EMD-25578	EMD-25576	EMD-25579	EMD-25575
**Map resolution 0.143 FSC**	6.5Å	6.2Å	8.6Å	7.8Å	5.9Å
**Density Modified Resolution 0.5Ref**	n/a	4.2Å	n/a	n/a	6.0Å
**Symmetry Imposed**	C3	D3	C1	C3	D4
**Number of particles**	25,437	33,479	73,042 (D3) 57,764 (C1)	16,244	50,686
**Refinement**					
**Map Sharpening B Factor**	$-330Å^2$	$-284Å^2$	$-471Å^2$	$-503Å^2$	$-430Å^2$

Table S3: Cryo-EM data collection and Refinement statistics.

Movie S1: 3D Variability analysis (3DVA) first component of the D3-C5 design. 3DVA was done in cryosparc v2 after D3 symmetry expansion while using default processing parameters and a 5Å low-pass filter. Variability results are displayed as the first trajectory component of variability calculated in simple mode as a 20 frame output, which are low-pass filtered to 5Å for clarity. Volume series is rendered as a movie using UCSF chimera. Movie shows rotation of the rotor through the volume series, as well as a transverse cross section of the central rotor and axle.

Movie S2: 3D Variability analysis (3DVA) second component of the D3-C5 design. 3DVA was done in cryosparc v2 after D3 symmetry expansion while using default processing parameters and a 5Å low-pass filter. Variability results are displayed as the second trajectory component of variability calculated in simple mode as a 20 frame output, which are low-pass filtered to 5Å for clarity. Volume series is rendered as a movie using UCSF chimera. Movie shows rotation of the rotor through the volume series, as well as a transverse cross section of the central rotor and axle.

Movie S3: 3D Variability analysis (3DVA) third component of the D3-C5 design. 3DVA was done in cryosparc v2 after D3 symmetry expansion while using default processing parameters and a 5Å low-pass filter. Variability results are displayed as the third trajectory component of variability calculated in simple mode as a 20 frame output, which are low-pass filtered to 5Å for clarity. Volume series is rendered as a movie using UCSF chimera. Movie shows rotation of the rotor through the volume series, as well as a transverse cross section of the central rotor and axle.

Movie S4: 3D Variability analysis (3DVA) of the D3-C3 design. 3DVA was done in cryosparc v2 after D3 symmetry expansion while using default processing parameters and a 5Å low-pass filter. Variability results are displayed as the first trajectory component of variability calculated in simple mode as a 20 frame output, which are low-pass filtered to 5Å for clarity. Volume series is rendered as a movie using UCSF chimera. Movie shows rotation of the rotor through the volume series, as well as a transverse cross section of the central rotor and axle.

Movie S5: 3D Variability analysis (3DVA) of the D8-C4 design. 3DVA was done in cryosparc v2 after D4 symmetry expansion while using default processing parameters and a 10Å low-pass filter. Variability results are displayed as the second trajectory component of variability calculated in simple mode as a 10 frame output, which are low-pass filtered to 15Å for clarity. Volume series is rendered as a movie using UCSF chimera. First and last frames of the second trajectory component were used as input for downstream refinement of distinct structures.
